# Nuclear egress of TDP-43 and FUS occurs independently of Exportin-1/CRM1

**DOI:** 10.1038/s41598-018-25007-5

**Published:** 2018-05-04

**Authors:** Helena Ederle, Christina Funk, Claudia Abou-Ajram, Saskia Hutten, Eva B. E. Funk, Ralph H. Kehlenbach, Susanne M. Bailer, Dorothee Dormann

**Affiliations:** 1 BioMedical Center (BMC), Cell Biology, Ludwig-Maximilians-University Munich, 82152 Planegg-Martinsried, Germany; 2 Graduate School of Systemic Neurosciences (GSN), 82152 Planegg-Martinsried, Germany; 3 Institute for Interfacial Engineering and Plasma Technology IGVP, University of Stuttgart, 70569 Stuttgart, Germany; 4 Frauenhofer Institute for Interfacial Engineering and Biotechnology, 70569 Stuttgart, Germany; 5 BioMedical Center (BMC), Biochemistry, Ludwig-Maximilians-University Munich, 81377 Munich, Germany; 6 Department of Molecular Biology, Faculty of Medicine, GZMB, Georg-August-University Göttingen, Humboldtallee 23, 37073 Göttingen, Germany; 7 Munich Cluster for Systems Neurology (SyNergy), 81377 Munich, Germany

## Abstract

TDP-43 and FUS are nuclear proteins with multiple functions in mRNA processing. They play key roles in ALS (amyotrophic lateral sclerosis) and FTD (frontotemporal dementia), where they are partially lost from the nucleus and aggregate in the cytoplasm of neurons and glial cells. Defects in nucleocytoplasmic transport contribute to this pathology, hence nuclear import of both proteins has been studied in detail. However, their nuclear export routes remain poorly characterized and it is unclear whether aberrant nuclear export contributes to TDP-43 or FUS pathology. Here we show that predicted nuclear export signals in TDP-43 and FUS are non-functional and that both proteins are exported independently of the export receptor CRM1/Exportin-1. Silencing of Exportin-5 or the mRNA export factor Aly/REF, as well as mutations that abrogate RNA-binding do not impair export of TDP-43 and FUS. However, artificially enlarging TDP-43 or FUS impairs their nuclear egress, suggesting that they could leave the nucleus by passive diffusion. Finally, we found that inhibition of transcription causes accelerated nuclear egress of TDP-43, suggesting that newly synthesized RNA retains TDP-43 in the nucleus, limiting its egress into the cytoplasm. Our findings implicate reduced nuclear retention as a possible factor contributing to mislocalization of TDP-43 in ALS/FTD.

## Introduction

The RNA-binding proteins TDP-43 (TAR DNA-binding protein of 43 kDa) and FUS (Fused in sarcoma) have become infamous over the past years as being the main culprits in two fatal neurodegenerative diseases, ALS (amyotrophic lateral sclerosis) and FTD (frontotemporal dementia). ALS is characterized by a progressive degeneration of motor neurons, which causes muscle weakness and eventually complete muscle paralysis. ALS patients typically die due to respiratory failure, usually 3–5 years after disease onset^1^. In FTD, a progressive degeneration of the frontal and temporal cortex leads to behavioral or language dysfunction. Eventually patients show severe cognitive impairment and die typically 7–10 years after disease onset^2^. ALS and FTD belong to the same disease spectrum and are thought to have a similar molecular cause, namely mislocalization and aggregation of RNA-binding proteins and, consequently, defective mRNA processing^3^.

TDP-43 and FUS are ubiquitously expressed proteins that belong to the family of heterogenous nuclear ribonucleoproteins (hnRNPs). Their main site of localization is the nucleus, where they bind to gene promotors or long introns of pre-mRNAs and regulate transcription or splicing, respectively^3–7^. They also play a role in miRNA biogenesis and are associated with lncRNAs in paraspeckles^7–9^. A small fraction of TDP-43 and FUS is found in the cytoplasm, where they regulate stability, transport and translation of certain mRNA targets^10–12^. In post-mortem brains of ALS and FTD patients, however, the localization of TDP-43 or, less frequently, FUS is dramatically altered: TDP-43 or FUS are lost from the nucleus of many neurons and glial cells and accumulate in large cytoplasmic protein aggregates, also called inclusions^13–15^. Occasionally, cells that have lost TDP-43 or FUS from the nucleoplasm also show intranuclear TDP-43 or FUS inclusions^15,16^, although this is much more rarely seen than cytoplasmic TDP-43 or FUS inclusions. On a functional level, this is thought to cause a loss of their normal mRNA processing functions. Moreover, TDP-43 or FUS aggregates are thought to gain novel toxic functions, e.g. due to aberrant protein/RNA interactions or altered mRNP granule dynamics^12,17^.

Research over the past few years has provided strong evidence that nuclear import defects contribute to the nuclear loss and cytoplasmic accumulation of TDP-43 and FUS and to ALS and FTD pathogenesis^18–20^. First, genetic mutations that alter or truncate the nuclear localization signal (NLS) of FUS and thus cause impaired nuclear import of FUS, cause familial ALS^21–24^ or motor neuron degeneration in mice^25–27^. Second, FTD patients with TDP-43 aggregates were shown to have reduced cortical levels of Exportin-2 (CAS)^28^. This Exportin re-exports the nuclear import receptor Importin α into the cytoplasm and therefore is required for proper Importin α/β-dependent nuclear import^29^. TDP-43 is imported into the nucleus by Importin α/β^28,30^, hence reduced Exportin-2 levels impair its nuclear import^28^. Third, the most common genetic cause of familial ALS and FTD, a hexanucleotide (GGGGCC) repeat expansion in the *C9orf72* gene, is thought to functionally compromise the nuclear transport machinery, as several components involved in protein import, protein export as well as mRNA export are strong genetic modifiers of *C9orf72* repeat-associated toxicity^31–35^. Consequently, enhancing nuclear import of TDP-43 and FUS could be a promising therapeutic approach, but will most likely be very hard to implement.

An alternative therapeutic approach could be to curb nuclear export of TDP-43 and FUS, in order to compensate for poor nuclear import and to restore normal nuclear TDP-43 and FUS levels. Inhibition of nuclear export as a therapeutic strategy has already been tested in preclinical models of *C9orf72*- and TDP-43-associated ALS and FTD: Here, specific inhibitors of the nuclear export receptor CRM1 (Exportin-1), KPT-276 and KPT-335, alleviated *C9orf72* repeat-mediated neurodegeneration in the *drosophila* eye^33^ and reduced TDP-43 overexpression-induced cell death in cortical neurons^36^, respectively. In another study, the CRM1 inhibitors KPT-276 and KPT-350 were shown to protect against axonal damage in preclinical models of demyelination and glutamate-induced neurotoxicity^37^, although the underlying mechanisms are not well understood.

CRM1 exports nuclear proteins that contain a so-called leucine-rich nuclear export signal (NES), which contains four closely spaced hydrophobic residues (Φ) and follows the consensus sequence Φx_2–3_Φx_2–3_ΦxΦ^38,39^. In the presence of RanGTP in the nucleus, CRM1 directly binds such NESs with its cargo binding site and transports the NES-containing cargo across nuclear pore complexes (NPCs) into the cytoplasm^40,41^. Here, RanGAP promotes conversion of RanGTP into RanGDP, which leads to dissociation of the CRM1-cargo complex. Whether CRM1 also recognizes TDP-43 and FUS and mediates active nuclear export of the two proteins is unknown. So far, a CRM1-dependent NES has been predicted in the RRM2 domain of TDP-43 and mutation of key hydrophobic residues were shown to result in nuclear aggregation of TDP-43^30^. A leucine-rich NES has also been predicted in the RRM domain of FUS^23,42^ and deletion of this putative NES was shown to suppress toxicity associated with NLS mutant versions of FUS^43^. Despite these interesting observations, functionality of the predicted NESs has not been tested in *bona fide* nuclear export assays.

In this study we used the interspecies heterokaryon assay as a nuclear export assay to address whether predicted CRM1-dependent NESs in TDP-43 and FUS are functional and whether pharmacological inhibition or silencing of CRM1 blocks nuclear export of TDP-43 and FUS. As our results demonstrate CRM1-independent nuclear egress of TDP-43 and FUS, we addressed whether RNA-binding, the mRNA export machinery or the export receptor Exportin-5 are required for nuclear export of TDP-43 or FUS. As this was not the case, we engineered enlarged versions of TDP-43 and FUS to test whether nuclear egress of TDP-43 and FUS occurs by passive diffusion rather than active receptor-mediated export. Finally, using biochemical extraction and fluorescence loss in photobleaching (FLIP) experiments, we obtained support for the hypothesis that newly synthesized RNA anchors TDP-43 in the nucleus and limits its egress into the cytoplasm. Our results raise the possibility that transcriptional inhibition or defective RNA-binding may accelerate diffusion of TDP-43 out of the nucleus and thus may contribute to cytoplasmic mislocalization of TDP-43 in ALS and FTD patients.

## Results

### Predicted CRM1-dependent NESs in TDP-43 and FUS are not functional

**Figure 1:**
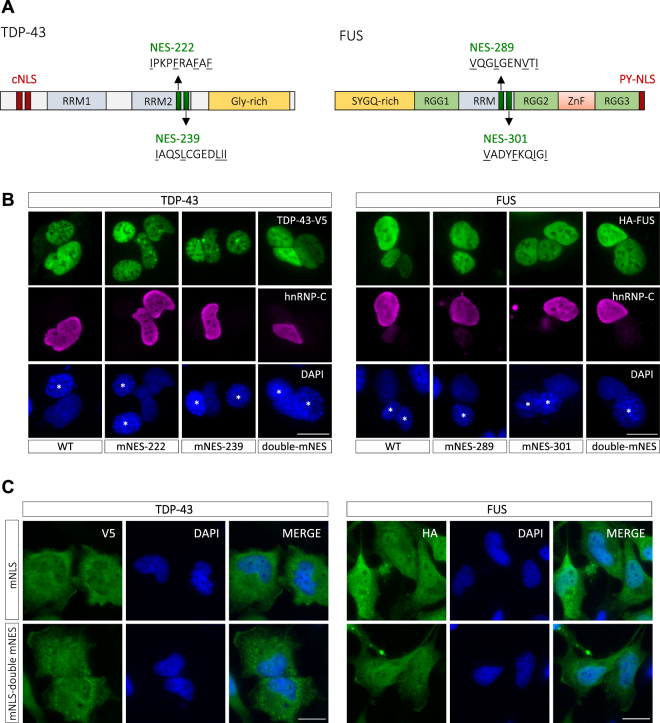
Predicted NESs of TDP-43 and FUS are non-functional. (**A**) Schematic diagrams of TDP-43 and FUS. TDP-43 contains an internal classical bipartite nuclear localization signal (cNLS), whereas FUS contains a proline-tyrosine (PY)-NLS at the C-terminus (in red). For each protein, two putative CRM1-dependent NESs (in green, key hydrophobic amino acids underlined) were predicted using bioinformatic NES prediction tools. They are localized within RNA-recognition motif (RRM) domains of TDP-43 and FUS. (**B**) Heterokaryon assay performed to analyze nuclear export of TDP-43 and FUS. HeLa cells expressing the indicated V5-tagged TDP-43 or HA-tagged FUS constructs were fused with mouse embryonic fibroblasts and the resulting heterokaryons were incubated for 2 h (TDP-43) and 5 h (FUS), respectively, in the presence of cycloheximide. Cells were stained with a V5- or HA-specific antibody (green), a human hnRNP-C-specific antibody to visualize a non-shuttling control protein in human nuclei (magenta) and DAPI as a nucleic acid stain (blue). The appearance of TDP-43 and FUS in the murine nucleus (marked with an asterisk in the DAPI channel) indicates that both proteins undergo nuclear export, in contrast to the non-shuttling control protein hnRNP-C. Mutation of key hydrophobic amino acids in one or both predicted NESs (mNES or double mNES) does not abrogate nuclear export, demonstrating that the predicted NESs are not necessary for TDP-43 or FUS export. Scale bars: 20 μm. (**C**) HeLa cells were transiently transfected with V5-tagged TDP-43 or HA-tagged FUS constructs carrying a mutated NLS (mNLS) or both a mutated NLS and mutations in the predicted NESs (mNLS-double mNES). Cells were stained with a V5- or HA-specific antibody (green) and DAPI (blue) and localization of mutant proteins was examined by fluorescence microscopy. Scale bars: 20 μm.

Using bioinformatic NES prediction tools (NES finder 0.2 and NetNES 1.1 Server), we identified two putative CRM1-dependent NESs in each TDP-43 (at positions 222 and 239) and FUS (at positions 289 and 301) (Fig. 1A). NES-239 (IAQSLCGEDLII) in TDP-43 and NES-289 (VQGLGENVTI) in FUS have been previously predicted as CRM1-dependent NESs^23,30^, however their activity has not been verified in *bona fide* nuclear export assays. NES-222 (IPKPFRAFAF) in TDP-43 and NES-301 (VADYFKQIGI) in FUS, which also fulfill the consensus criteria for recognition by CRM1, have not been predicted and studied before. To address the functionality of these predicted NESs, we introduced mutations that should abrogate CRM1-binding, by exchanging two key hydrophobic residues of one or both predicted NESs for alanine (A) (Fig. S1A). When expressed in HeLa cells, all mutant proteins showed the same cellular localization as wild-type (WT) TDP-43 and FUS (Fig. S1B). We then examined nuclear export of WT and NES-mutant versions (mNES) of TDP-43 and FUS in the interspecies heterokaryon assay, a well-established nuclear export assay^44^. In this assay, cells from two different species (e.g. human and mouse) are chemically fused to generate hybrid cells, so-called interspecies heterokaryons (see schematic diagram in Fig. S2A). If the nuclear protein-of-interest shuttles between the nucleus and cytoplasm, it will enter the other nucleus and accumulate there over time. The assay is carried out in the presence of a protein synthesis inhibitor to exclude that the signal in the receptor nucleus comes from newly synthesized protein in the cytoplasm. We expressed V5-tagged TDP-43 or HA-tagged FUS (WT or NES-mutant versions) in human HeLa cells and fused them with mouse embryonic fibroblasts to examine whether shuttling from the human to the mouse nucleus occurred in interspecies heterokaryons. TDP-43 accumulated in mouse nuclei (marked by an asterisk) over the course of 2 hours, whereas FUS shuttled at a slightly slower rate and prominently appeared in mouse nuclei after 5 hours (Fig. S2B). Similar to the WT proteins, both single and double NES mutants of TDP-43 and FUS accumulated in mouse nuclei 2–5 hours post-fusion, whereas the non-shuttling control protein hnRNP-C was only detectable in human nuclei (Fig. 1B). Thus, the predicted NESs in TDP-43 and FUS do not appear to be required for nuclear export of TDP-43 and FUS. To substantiate this finding, we introduced the double NES mutation into TDP-43 and FUS constructs carrying a mutated nuclear localization signal (mNLS). These mutant proteins show impaired nuclear import and hence a partial cytosolic mislocalization^45^. We reasoned that if one of the two predicted NESs would be functional, mutating them should shift the equilibrium towards a more nuclear localization. However, the double NES mutation did not alter the nucleocytoplasmic localization of NLS mutant TDP-43 and FUS (Fig. 1C). To further address functionality of the predicted NESs, we fused them to the C-terminus of EGFP, which is localized in both the nucleus and the cytoplasm, with a predominant nuclear localization^46^. We reasoned that if the predicted NESs would be functional, the fusion proteins should be recognized by CRM1 and actively exported to the cytoplasm. However, none of the EGFP-NES fusion proteins showed a more cytoplasmic localization when compared to EGFP alone (Fig. S2C). Together, our data demonstrate that TDP-43 and FUS can leave the nucleus, but that the predicted CRM1-dependent NESs are neither necessary nor sufficient for nuclear export and hence are not functional NESs.

### Nuclear export of TDP-43 and FUS is independent of CRM1

**Figure 2:**
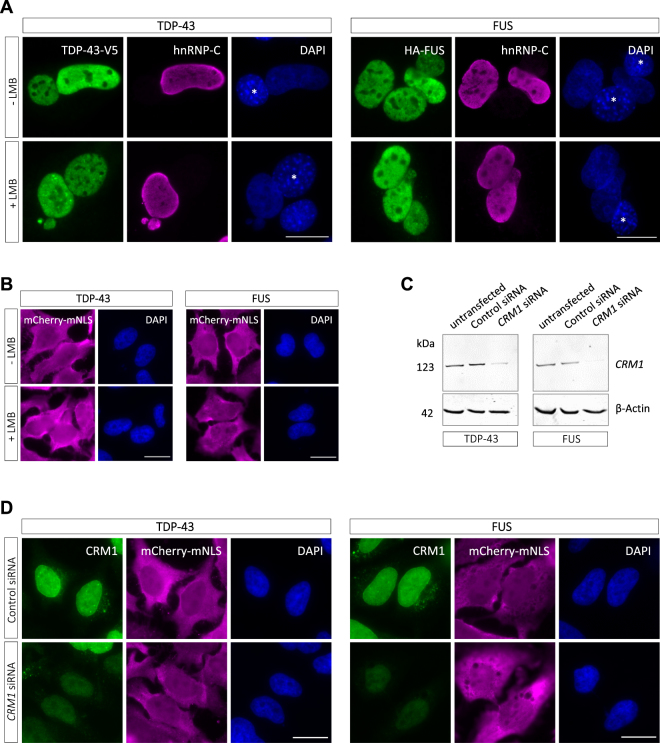
Nuclear export of TDP-43 and FUS is CRM1-independent. (**A**) Heterokaryon assay performed to analyze nuclear export of TDP-43 and FUS with or without CRM1 inhibition. 20 nM Leptomycin B (LMB) was added to heterokaryons directly after fusion and cells were incubated for 2 h (TDP-43) and 3 h (FUS), respectively, in the presence of cycloheximide. Cells were stained with a V5- or HA-specific antibody (green), a human hnRNP-C-specific antibody (magenta) and DAPI as a nucleic acid stain (blue). LMB does not inhibit shuttling of TDP-43 and FUS from human to mouse nuclei (marked with an asterisk in the DAPI channel), demonstrating that export of TDP-43 and FUS is CRM1-independent. Scale bars: 20 μm. (**B**) Hela cells stably expressing mCherry-tagged TDP-43 and FUS with mutated NLS (mNLS) were treated with LMB (20 nM) for 2.5 hours. LMB treatment does not cause a nuclear accumulation of NLS mutant proteins. Scale bars: 20 μm. (**C**) and (**D**) HeLa cells stably expressing mCherry-TDP-43-mNLS or mCherry-FUS-mNLS were transfected with control or *CRM1*-specific siRNA. 3 days post-transfection, CRM1 levels in total cell lysates were analyzed by Western blotting, β-actin served as a loading control. (**C**) In parallel, cells were stained with a CRM1-specific antibody (green), a mCherry-specific antibody (magenta) and DAPI and were imaged by fluorescence microscopy. (**D**) Cells with reduced CRM1 levels do not show a nuclear accumulation of mCherry-tagged NLS mutant TDP-43 or FUS. Scale bars: 20 μm.

Even though the predicted CRM1-dependent NESs are non-functional, export of TDP-43 and FUS could still be mediated by CRM1, either via a non-canonical NES not identified by the above mentioned NES prediction algorithms, or via a binding partner that contains a CRM1-dependent NES. We therefore tested whether pharmacological inhibition of CRM1 with leptomycin B (LMB), a well-known CRM1-specific inhibitor^47^, impairs nuclear export of TDP-43 or FUS in the heterokaryon assay. As a positive control for LMB activity, localization of p62/SQSTM1, a known CRM1 export substrate^48^, was analyzed in parallel. After LMB treatment for 2 hours, p62 strongly relocalized from the cytoplasm to the nucleus (Fig. S3A), demonstrating that LMB at the given concentration efficiently inhibited CRM1-dependent protein export in HeLa cells. In contrast, treatment of heterokaryons with LMB did not impair shuttling of TDP-43 and FUS from the human to the mouse nucleus (Fig. 2A), demonstrating that nuclear export of TDP-43 and FUS occurs independently of CRM1.

To further substantiate this finding, we made use of HeLa cells stably expressing mCherry-tagged TDP-43 or FUS with a mutated NLS (mNLS), which show a partial cytosolic mislocalization^45^. We reasoned that if TDP-43 and FUS would be exported via CRM1, LMB treatment or silencing of CRM1 with siRNA should shift the equilibrium towards a more nuclear localization. However, LMB treatment for 2.5 h did not alter the subcellular distribution of NLS-mutant TDP-43 or FUS (Fig. 2C). Similarly, 3 days after transfection with CRM1-specific siRNA, which efficiently reduced CRM1 protein levels (Fig. 2D,E), NLS-mutant TDP-43 and FUS showed the same predominantly cytosolic localization as in control siRNA-treated cells (Fig. 2E). Moreover, combining CRM1 knockdown and LMB treatment did not result in a more nuclear accumulation of NLS-mutant TDP-43 or FUS (Fig. S3B). Taken together, this demonstrates that TDP-43 and FUS are not primarily exported via CRM1 and hence are capable of utilizing alternative nuclear export routes.

### TDP-43 and FUS are exported independently of Exportin-5

As overexpression of the yeast homologue of Exportin-5, MSN5, was shown to enhance TDP-43 toxicity in yeast^49^, we next tested whether Exportin-5 (*XPO5*) is involved in nuclear export of TDP-43 and FUS. To this end, we used siRNA to silence *XPO5* in HeLa cells stably expressing NLS-mutant TDP-43 and FUS. *XPO5* knockdown was efficient, as demonstrated by immunoblotting (Fig. S4A) or co-staining with an Exportin-5-specific antibody (Fig. S4B), respectively. Nevertheless, NLS-mutant TDP-43 or FUS did not show an altered subcellular localization upon XPO5 silencing (Fig. S4B), demonstrating that Exportin-5 is not responsible for transporting TDP-43 and FUS out of the nucleus.

### Nuclear export of TDP-43 and FUS does neither require RNA-binding nor the mRNA export machinery

**Figure 3:**
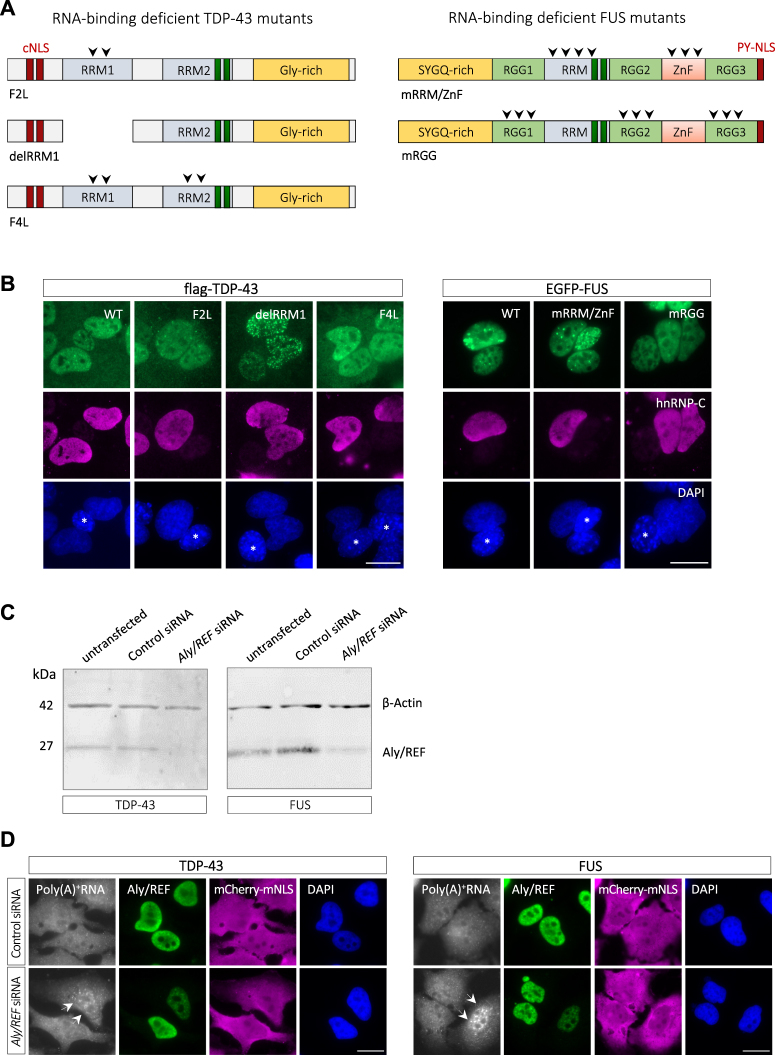
Nuclear export of TDP-43 and FUS does not require RNA-binding and is independent of the mRNA export machinery. (**A**) Schematic diagram of RNA-binding deficient TDP-43 and FUS mutants. 2FL = F147L/F149L; 4FL = F147L/F149L/F229L/F231L; mRRM/mZnF = 7 point mutations in the RRM domain/6 point mutations in the zinc finger (see methods for details); mRGG = all Rs in RGG motifs were exchanged for K. (**B**) The indicated flag-tagged TDP-43 or EGFP-FUS constructs were transiently transfected into HeLa cells and nuclear export was examined in the interspecies heterokaryon assay. Heterokaryons were incubated for 2 h (TDP-43) and 3 h (FUS), respectively, in the presence of cycloheximide and localization of TDP-43 or FUS proteins were visualized by flag immunostaining or direct EGFP fluorescence (green), respectively, hnRNP-C immunostaining (magenta) and DAPI (blue). Both wild-type (WT) and RNA-binding deficient mutant versions of TDP-43 and FUS shuttle from human to mouse nuclei (marked with an asterisk in the DAPI channel). Scale bars: 20 μm. (**C**) and (**D**) HeLa cells stably expressing mCherry-TDP-43-mNLS or mCherry-FUS-mNLS were transfected with control or *Aly/REF*-specific siRNA. 3 days post-transfection, Aly/REF levels in total cell lysates were analyzed by Western blotting, β-actin served as a loading control (**C**). In parallel, cells were processed for fluorescence *in situ* hybridization (FISH) and immunocytochemistry to visualize poly(A) + mRNA (white), Aly/REF (green), mCherry-TDP-43/FUS-mNLS (magenta) and were stained with DAPI (blue) (**D**). Cells with reduced Aly/REF levels show a nuclear accumulation of poly(A) + mRNA (arrows), however no nuclear accumulation of mCherry-tagged NLS mutant TDP-43 or FUS. Scale bars: 20 μm.

As TDP-43 and FUS are RNA-binding proteins involved in pre-mRNA splicing of numerous target RNAs^4,6^, it seems possible that they are exported along with fully spliced mRNAs via the mRNA export machinery. To address this hypothesis, we analyzed nuclear export of RNA-binding-deficient TDP-43 and FUS mutants (Fig. 3A) in the heterokaryon assay. In TDP-43, the predominant RNA-binding domain is RRM1, and deletion of this domain (ΔRRM1) as well as point mutation of key phenylalanines responsible for direct RNA interactions of RRM1 (2FL) and RRM1/RRM2 (4FL) were previously shown to abrogate RNA-binding and TDP-43′s autoregulation and RNA-splicing activity^50,51^. In FUS, we mutated residues previously shown to be crucial for RNA-binding of the RRM domain^52,53^ and a homologous RanBP2-type zinc finger (mRRM/mZnF)^54^. In another construct, we exchanged all RGG motifs to KGG motifs (mRGG), as RGG mutations were recently shown to abrogate high affinity RNA-binding of FUS in cells^55^. We found that all mutant proteins were still able to shuttle from the human to the mouse nucleus of heterokaryons within 2–3 h (Fig. 3B), indicating that mRNA-binding is not required for nuclear export of TDP-43 and FUS. To complement this finding, we silenced a key factor of the mRNA export machinery, Aly/REF, and examined whether this causes nuclear relocalization of NLS-mutant TDP-43 or FUS. Aly/REF is part of the TREX complex that recruits the TAP/p15 export receptor to processed mRNPs and allows their interaction with NPCs and passage through nuclear pores^56,57^. Aly/REF protein levels were efficiently reduced 3 days after siRNA transfection (Fig. 3C) and cells with reduced Aly/REF levels showed an accumulation of poly(A) + RNA in the nucleus (Fig. 3D), as previously reported^58^. Nevertheless, localization of NLS-mutant TDP-43 or FUS was indistinguishable in Aly/REF-depleted cells and control cells (Fig. 3D). This suggests that nuclear export of TDP-43 and FUS does not require a functional mRNA export machinery and occurs independently of bulk mRNA export.

### Artificial enlargement of TDP-43 and FUS impairs their nuclear egress

**Figure 4:**
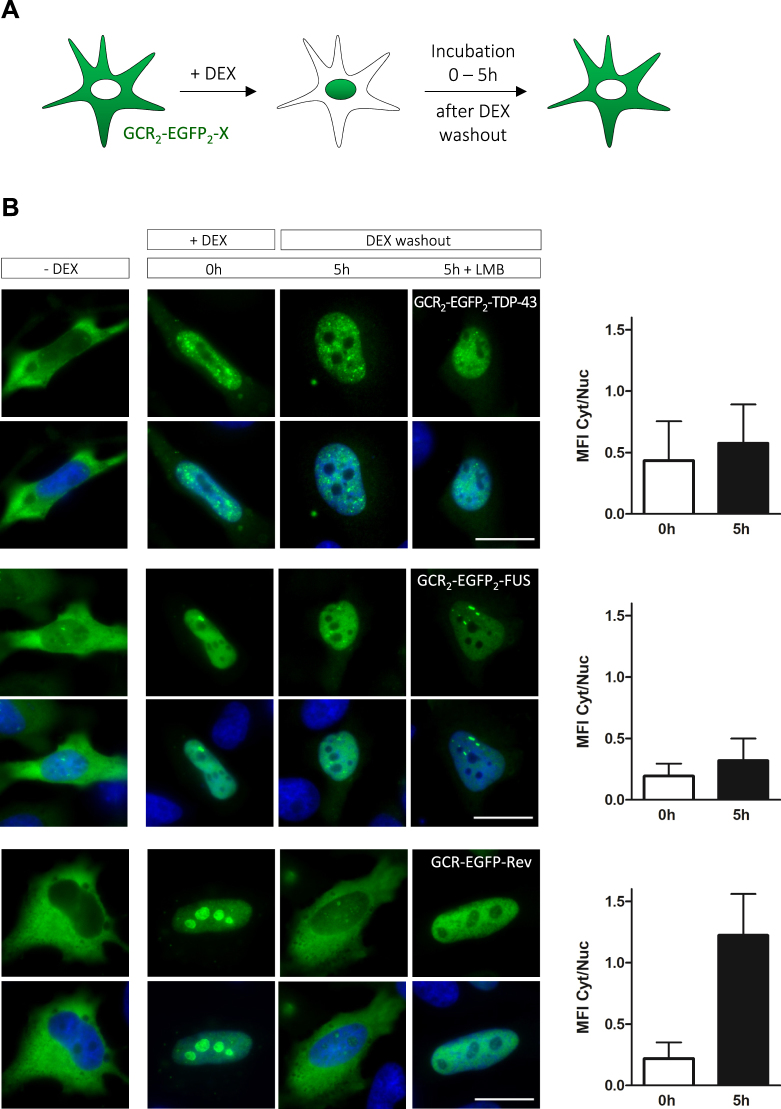
TDP-43 and FUS enlarged by ~120 kDa are poorly exported, suggesting that TDP-43 and FUS leave the nucleus by passive diffusion. (**A**) Schematic diagram of the hormone-induced nuclear transport assay. An NLS-containing protein-of-interest (X) is attached to the hormone-responsive domain of the glucocorticoid receptor (GCR). The GCR domains trap the fusion protein in the cytoplasm until dexamethasone addition (+DEX) releases trapping and induces rapid nuclear import. If the protein-of-interest contains an NES that mediates active nuclear export, rapid relocalization to the cytoplasm is observed after dexamethasone washout. (**B**) GCR_2_-EGFP_2_ (119 kDa) fused to TDP-43 or FUS were transiently expressed in HeLa cells and nuclear import was induced by DEX addition (+DEX). Localization of fusion proteins was examined at the indicated timepoints after DEX washout in the absence or presence of leptomycin B (LMB). Even 5 h after DEX removal, GCR_2_-EGFP_2_-TDP-43 or -FUS remain predominantly nuclear and only small amounts are observed in the cytoplasm. In contrast, GCR-EGFP-Rev (73 kDa), which contains a functional CRM1-dependent NES, is efficiently exported after DEX washout and its export is blocked by LMB treatment, demonstrating CRM1 dependence. Scale bars: 20 μm. Bar graphs to the right show MFI Cyt/Nuc ratios for the 0 h and 5 h timepoints (40 cells/condition from one representative experiment out of three), error bars indicate SD.

As TDP-43 and FUS appear to be exported independently of major protein export receptors (CRM1, Exportin-5) and the bulk mRNA export machinery, we next tested the hypothesis that the two proteins exit the nucleus by passive diffusion. To test our hypothesis directly, we adapted the hormone-inducible nuclear transport assay from Love *et al*., in which the hormone-binding domain of the glucocorticoid receptor (GCR) is fused to an NLS-containing protein-of-interest^59^. The GCR domain traps the fusion protein in the cytoplasm until addition of a steroid hormone, e.g. dexamethasone (DEX), triggers nuclear import; removal of the steroid hormone induces nuclear re-export (Fig. 4A). We fused two GCR domains (65 kDa total) and two EGFPs (54 kDa total) (GCR_2_-EGFP_2_) to the N-terminus of TDP-43 and FUS and thus enlarged them by 119 kDa, creating fusion proteins of 162 kDa and 172 kDa, respectively. If TDP-43 and FUS indeed exit the nucleus by passive diffusion, these large fusion proteins should leave the nucleus very slowly, as the rate of passive macromolecular diffusion through NPCs strongly decreases with increasing molecular weight^60,61^. In the absence of dexamethasone, GCR_2_-EGFP_2_-TDP-43 and -FUS were largely cytosolic, but rapidly translocated into the nucleus upon dexamethasone addition (Fig. 4B). After washout of dexamethasone, they remained largely nuclear over the course of 5 hours, whereas the well-characterized CRM1 cargo Rev^62,63^ fused to GCR-EGFP showed efficient re-export during the same time period (Fig. 4B, lower panels). Cytosolic relocalization of the GCR-EGFP-Rev fusion protein (73 kDa) occurred primarily by active CRM1-mediated nuclear export and not by passive diffusion, as it was blocked by LMB (Fig. 4B), thus serving as an appropriate positive control for receptor-mediated active export. These data suggest that nuclear export of TDP-43 and FUS becomes highly inefficient when the proteins are enlarged. This behavior is typically observed for cargoes that permeate NPCs by passive diffusion^64^. Thus, at least in HeLa cells, TDP-43 and FUS appear to egress from the nucleus primarily by passive diffusion rather than active, receptor-mediated export.

**Figure 5:**
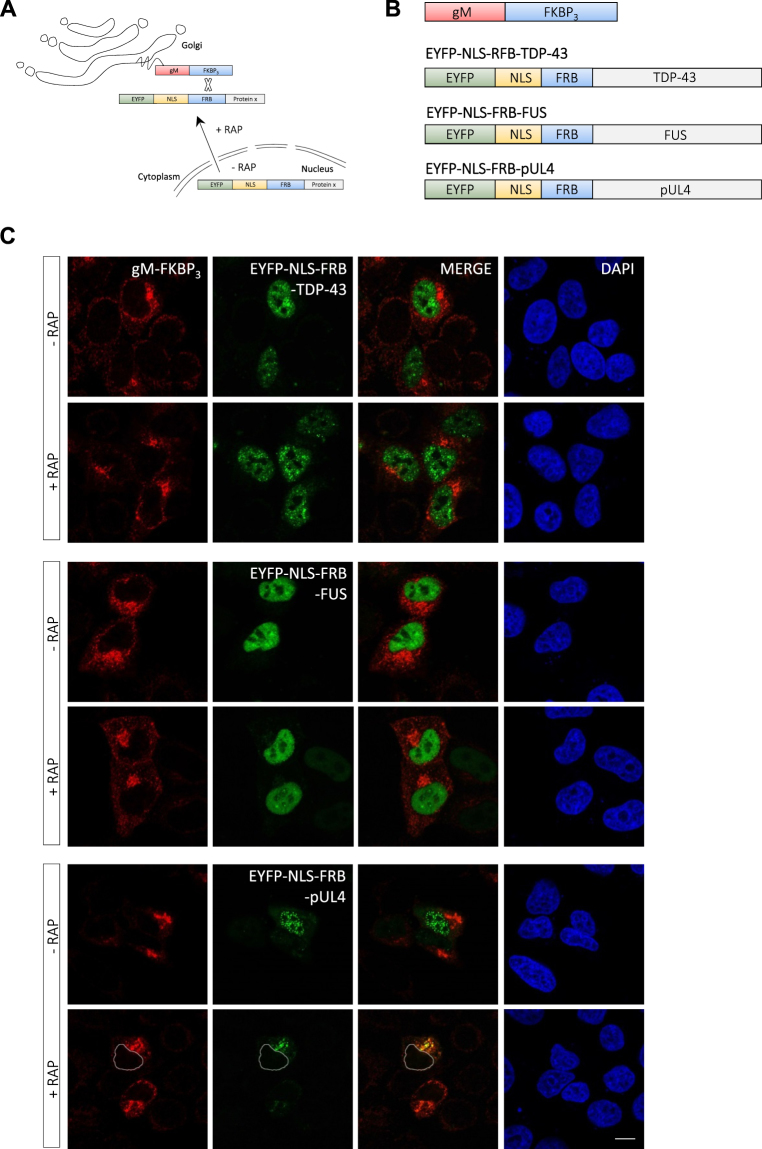
TDP‐43 and FUS are not actively exported in the NEX‐TRAP nuclear export assay. (**A**) Schematic diagram of the NEX-TRAP nuclear export assay. (**B**) Schematic diagram of fusion proteins used in the NEX-TRAP assay. (**C**) HeLa cells were co-transfected with a gM-FKBP_3_ construct and constructs encoding either pEYFP-NLS-FRB-TDP-43 or pEYFP-NLS-FRB-FUS. As a positive control, a plasmid encoding EYFP-NLS-FRB-pUL4 (a known CRM1 cargo) was used. 20 h post-transfection, cells were treated with the protein synthesis inhibitor anisomycin for 10 min and incubated for another 2 h in the presence or absence of rapamycin (RAP). Protein localization was visualized by anti-gM immunostaining (red); EYFP-tagged proteins were visualized directly (green); nuclei were visualized by DAPI staining (blue) and are marked by dashed lines. Scale bars: 10 μm.

To further substantiate this conclusion, we utilized the NEX-TRAP (Nuclear Export Trapped by RAPamycin) nuclear export assay, which is based on rapamycin-induced dimerization of FRB (FK506-rapamycin (FR)-binding domain) and FKBP (FK506-binding protein-12)^65^ (see Fig. 5A for a schematic diagram). 3 FKBP domains are exposed on the cytoplasmic side of the trans Golgi network (TGN) by fusion to an integral membrane protein (gM-FKBP_3_) and serve as a cytoplasmic reporter (Fig. 5A). The potential nuclear export cargo is fused to FRB, EYFP (for direct visualization) and three SV40 NLSs (for efficient nuclear import). Without rapamycin, EYFP-NLS-FRB fusion proteins with export activity continuously shuttle between the nucleus and cytoplasm, whereas addition of rapamycin induces dimerization of FRB and FKBP and traps the shuttling protein at the TGN. If the fusion protein does not contain an NES that mediates active nuclear export, trapping at the TGN does not occur. We fused EYFP-NLS-FRB (43 kDa) to the N-terminus of TDP-43 or FUS (Fig. 5B) and tested whether rapamycin induces trapping of the fusion proteins on the gM-FKBP_3_ reporter. However, both in the absence and presence of rapamycin (for 2 h), the TDP-43 and FUS fusion proteins were localized in the nucleus and no relocalization and trapping in the cytoplasm by TGN-resident gM-FKBP_3_ was observed (Fig. 5C, upper and middle panels). In contrast, our positive control, the NES-containing HSV-1 protein UL4^65,66^ (21 kDa) fused to EYFP-NLS-FRB was readily depleted from the nucleus and co-localized with gM-FKBP_3_ at the TGN upon rapamycin addition (Fig. 5C, lower panels). Although we cannot exclude that the SV40 NLSs present in the EYFP-NLS-FRB tag is simply too strong and masks export of the TDP-43 and FUS fusion proteins by causing immediate re-import, the results from the NEX-TRAP assay support the idea that TDP-43 and FUS lack an efficient NES that mediates active nuclear export.

Together, our results imply that TDP-43 and FUS, at least in HeLa cells, leave the nucleus predominantly by passive diffusion, as artificial enlargement of both proteins strongly impaired their nuclear egress, which is compatible with recent models of passive diffusion through NPCs^60,61,67^.

### Newly synthesized RNA retains TDP-43 in the nucleus and limits its diffusion into the cytoplasm

**Figure 6:**
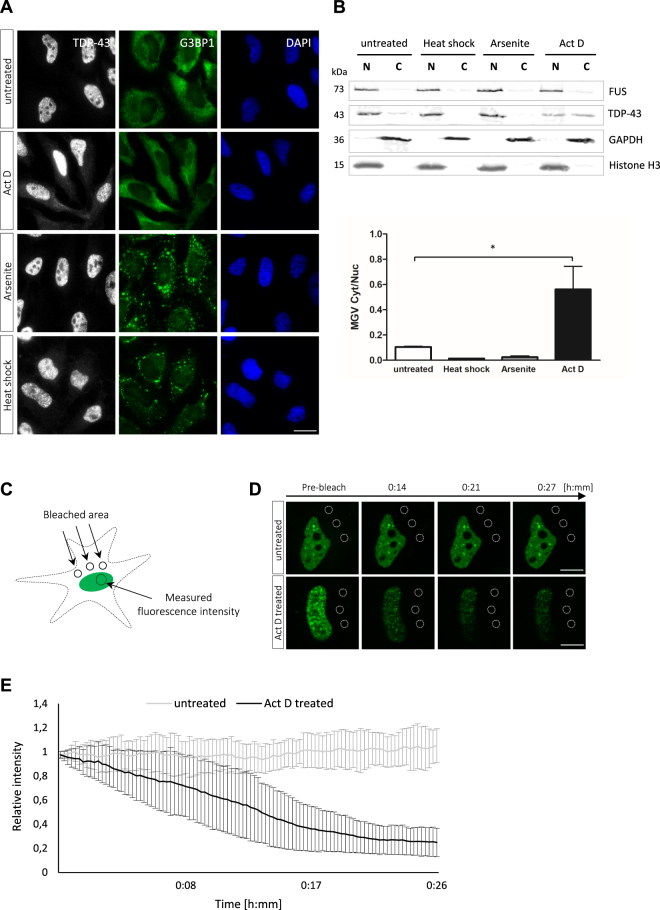
Inhibition of RNA synthesis causes accelerated nuclear egress of TDP-43. (**A**) HeLa cells were either left untreated or treated with Actinomycin D (Act D, 5 µg/ml for 3 h), arsenite (0.5 mM for 30 min) or heat shock (44 °C for 1 h) and localization of TDP-43 and stress granule formation were examined after immunostaining with antibodies specific for TDP-43 (white), the stress granule marker G3BP1 (green) and DAPI staining (blue). Act D treatment causes a slight relocalization of TDP-43 to the cytoplasm in the absence of stress granule formation. Other stress treatments induce stress granules without inducing TDP-43 relocalization. Scale bar: 20 µm. (**B**) HeLa cells treated as indicated in A were subjected to hypotonic lysis and separated into a nuclear fraction (N) containing insoluble nuclear proteins and a cytoplasmic fraction (**C**) containing soluble nuclear and cytoplasmic proteins. Fractions were analyzed by SDS-PAGE and immunoblotting with TDP-43- or FUS-specific antibodies. GAPDH served as a cytosolic marker, Histone H3 as a nuclear marker protein. While TDP-43 is predominantly in the nuclear fraction of untreated, arsenite-treated and heat shocked cells, it is extracted from nuclei into the cytoplasmic fraction after Act D treatment. FUS remains predominantly in the nuclear fraction upon inhibitor treatment. Bar graph shows a quantification of TDP-43 immunoblot signals. Mean grey value (MGV) of bands in cytoplasmic/nuclear fraction of three independent experiments are shown, error bars represent SEM; *p-value ≤ 0.05 by one-way Anova with Dunnett’s multiple comparison test. (**C**) Schematic diagram of FLIP assay used to measure nuclear export of EGFP-TDP-43 in living HeLa cells. Three defined areas in the cytoplasm are repeatedly bleached, while fluorescence loss of the EGFP-signal in the nucleus is monitored over time using spinning disc confocal miscroscopy. (**D**) Representative images of untreated and 3 h Act D-treated EGFP-TDP-43-expressing cells recorded during FLIP analysis. Act D-treatment leads to accelerated loss of the nuclear EGFP-signal, whereas the nuclear EGFP-signal remains constant in untreated cells over the course of ~30 min. Scale bars: 10 μm. (**E**) Rate of nuclear egress of EGFP-TDP-43 measured by FLIP analysis in untreated and 3 h Act D-treated HeLa cells. Relative fluorescence intensity of the nuclear EGFP-signal is stable in untreated cells, but decreases over time in Act D-treated cells, demonstrating that transcriptional inhibition accelerates nuclear egress of TDP-43^101^.

As TDP-43 and FUS appear to leave the nucleus by passive diffusion, intranuclear interactions could limit or slow down nuclear egress of TDP-43 and FUS by nuclear retention. TDP-43 and FUS are known to bind to long introns of hundreds or thousands of pre-mRNAs and to regulate alternative splicing^3,5,6,68^. We therefore speculated that newly synthesized RNA could act as an anchor that retains TDP-43 and FUS in the nucleus and limits their diffusion through NPCs into the cytoplasm. To test this hypothesis, we treated HeLa cells with the transcriptional inhibitor actinomycin D (Act D) for 3 hours and examined subcellular localization and biochemical extractability of TDP-43 and FUS in comparison to untreated cells. Indeed, Act D treatment caused a slight cytosolic relocalization of TDP-43 (Figs 6A, S5B), consistent with a previous report^69^. Cytoplasmic relocalization of TDP-43 was not observed with stress treatments that induce stress granules, e.g. arsenite treatment or heat shock, and no G3BP1-positive stress granules were observed in Act D-treated cells (Fig. 6A), demonstrating that cytoplasmic relocalization of TDP-43 upon Act D treatment is not due to stress granule formation. FUS remained predominantly nuclear after Act D treatment (Fig. S5A,B), however, cytoplasmic redistribution of FUS has previously been reported to occur under transcriptional inhibition^70^. Hence, it is possible that the anti-FUS antibody we used was not sensitive enough to detect small amounts of FUS in the cytoplasm, or that cytoplasmic FUS is very rapidly re-imported by its nuclear import receptor Transportin^21,71^. Upon hypotonic lysis and nuclear/cytoplasmic fractionation, TDP-43 was mostly restricted to the nuclear fraction in untreated, arsenite-treated and heat-shocked cells, but could be partially extracted from nuclei of Act D-treated cells (Fig. 6B). This suggests that newly synthesized RNA retains TDP-43 in the nucleus, whereas transcriptional inhibition causes TDP-43 to become soluble and extractable into the cytoplasmic fraction. A higher extractability from the nucleus was also observed for a mutant version of TDP-43 that is unable to bind to RNA (EGFP-TDP-4FL)^50^ (Fig. S5C). Nevertheless, EGFP-TDP-4FL was exclusively found in the nucleus, possibly because the previously described oligomerization/partitioning of the mutant protein into nuclear granules^69^ (Fig. S5D) precludes its diffusion across NPCs into the cytoplasm.

To examine whether transcriptional inhibition indeed accelerates nuclear egress of TDP-43 in intact and living cells, we treated EGFP-TDP-43-expressing HeLa cells with or without Act D for 3 hours and measured the loss of nuclear EGFP fluoresence upon repeated bleaching of defined areas in the cytoplasm (fluorescence loss in photobleaching, FLIP, see schematic diagram in Fig. 6C). In this assay, loss of nuclear EGFP fluorescence is primarily a measure of the nuclear export rate. While a minor contribution of re-import into the nucleus cannot entirely be excluded, it should be insignificant in this assay, as the cytoplasmic pool of EGFP-TDP-43 is very small and the entire cytoplasm is eventually bleached by repeated photobleaching of defined cytoplasmic areas^72^. Indeed, we observed a more rapid loss in nuclear EGFP fluorescence in Act D-treated cells compared to untreated cells (Fig. 6D,E), indicating that EGFP-TDP-43 leaves the nucleus more rapidly upon transcriptional inhibition. To exclude that loss of nuclear EGFP fluorescence was caused unspecifically by Act D treatment, e.g. due to reduced cell viability, we performed FLIP analysis in cells expressing EGFP-tagged histone H3.3. For this control protein, only a slight loss of nuclear EGFP fluorescence was observed in Act D-treated cells over the same time course (Fig. S6A,B). Thus, the much stronger loss of nuclear EGFP fluorescence observed for EGFP-TDP-43 most likely predominantly results from accelerated nuclear egress of TDP-43 upon transcriptional inhibition.

## Discussion

In our study we examined how TDP-43 and FUS leave the nucleus, as the nuclear export routes of the two shuttling hnRNPs have not been characterized so far. In particular, we sought to test the hypotheses that TDP-43 and FUS are exported by the nuclear export receptor CRM1 and that inhibition of CRM1 may be a therapeutic strategy to compensate for defective nuclear import of TDP-43 and FUS. Our results clearly demonstrate that TDP-43 and FUS leave the nucleus independently of CRM1 and that pharmacologic inhibition of CRM1 is unable to restore nuclear localization of TDP-43 and FUS when their nuclear import is impaired. Our findings are in line with quantitative proteomic studies that searched for proteins that bind to CRM1 in a Ran-GTP-dependent manner^73^ or shift their localization upon LMB treatment^74,75^. In these studies, TDP-43 and FUS were not among the CRM1-binding proteins or proteins that show elevated nuclear levels upon CRM1 inhibition. Our data suggest that CRM1 inhibitors (e.g. KPT-350) most likely exert their promising neuroprotective effects^33,36,37^ independently of TDP-43 and FUS. Instead, their beneficial effects must be due to inhibiting export of other CRM1 cargoes, e.g. Tau and NRF2 (nuclear factor, erythroid 2 like 2). Deciphering the neuroprotective mechanism of CRM1 inhibitors in different models of neurodegeneration will be an interesting task for the future.

We also tested whether previously predicted leucine-rich NESs in TDP-43 (NES-239 IAQSLCGEDLII)^30^ and FUS (NES-289 VQGLGENVTI)^23^ have NES activity using the heterokaryon nuclear export assay. We found that TDP-43 and FUS are able to leave the nucleus even when key hydrophobic residues in the predicted NES are exchanged for alanine. We cannot completely exclude that other functional CRM1-dependent NESs, not predicted by available NES prediction tools, are present in TDP-43 and FUS. However, we consider this highly unlikely, as we found that neither pharmacological CRM1 inhibition nor siRNA-mediated CRM1 silencing (or both combined) alter nuclear egress/localization of TDP-43 or FUS. For TDP-43, it was furthermore shown that TDP-43 localization is unaffected by CRM1 inhibition in primary rat neurons^76^, suggesting that our findings, at least for TDP-43, are not limited to cell lines, such as HeLa cells, but can be extended to neuronal cells.

In light of our finding, the question arises what causes nuclear aggregation of TDP-43 when the predicted NES-239 is mutated^30^, and why toxicity associated with TDP-43 or FUS overexpression is suppressed when the predicted NES is deleted^43,77,78^. Both predicted signals are located in RNA recognition motif (RRM) domains, hence it seems possible that the point mutations/deletion alter RRM folding and impair RNA-binding of TDP-43 or FUS. Loss of RNA-binding is expected to cause nuclear aggregation of TDP-43^69,79^ and to reduce TDP-43- or FUS-associated toxicity^52,80,81^. In direct support of the idea that mutations in the predicted “NES-239” of TDP-43 abrogate RNA-binding, this sequence was shown to be located within the hydrophobic core of the TDP-43 RRM2 domain^82^ and mutations in “NES-239” were found to disrupt TDP-43’s splicing activity^76^. In summary, we conclude that the previously predicted and commonly annotated NESs in TDP-43 and FUS are not functional. Mutations in these motifs do not affect nuclear egress of TDP-43 and FUS and hence are not suited to experimentally trap TDP-43 or FUS in the nucleus. Instead, they most likely impair RNA-binding and RNA processing functions of TDP-43 and FUS, which should be kept in mind when interpreting data generated with such “NES” mutant versions.

Besides addressing CRM1 as possible export receptor, we excluded that Exportin-5 or the mRNA export machinery solely mediate nuclear export of TDP-43 and FUS. We considered Exportin-5 as a possible candidate, as it has been genetically linked to TDP-43 toxicity^49^. Export along with mRNA appeared to be a likely scenario, as TDP-43 and FUS are well-known RNA binding proteins and FUS was found to be associated with CIP29, a component of the TREX mRNA export complex^83^. Overall, our data argue against a predominantly receptor-mediated nuclear export of TDP-43 and FUS, as artificial enlargement (with domains of 43 or 119 kDa, respectively) interfered with nuclear egress of both proteins. These data support the idea that TDP-43 and FUS leave the nucleus to a large extent by passive diffusion. It is well known that passive macromolecular diffusion through NPCs decreases strongly as macromolecules increase in size. Older studies reported a size threshold of 40–60 kDa^84–86^. However, more recent studies indicate that there is no firm size threshold, but rather a soft barrier to passive diffusion that gradually intensifies with increasing size of macromolecules^61,67^. Consistent with this model, smaller molecules passively permeate NPCs within minutes, whereas larger molecules (of up to 230 kDa) were found to permeate NPCs on the time scale of hours^60,61,64^. Nuclear egress of TDP-43 and FUS appears to follow this model of passive diffusion: Both proteins shuttle efficiently between nuclei of heterokaryon when they carry a small epitope tag (V5-TDP-43 or HA-FUS), but poorly exit the nucleus when tagged with larger domains (>43 kDa). This suggests that TDP-43 and FUS, at least in HeLa cells, leave the nucleus by passive diffusion rather than facilitated receptor-mediated export.

The notion that TDP-43 and FUS leave the nucleus by passive diffusion implies that any molecular interactions that (i) lead to higher molecular weight complexes of TDP-43 or FUS or (ii) trap TDP-43 or FUS on nuclear structures will reduce their nuclear egress and favor a predominantly nuclear localization. In the case of TDP-43, it has been recently reported that TDP-43 forms homo-oligomers (dimers, tetramers and higher oligomeric species) under physiological conditions, which antagonizes its pathological aggregation^87^. FUS also has been suggested to self-assemble and oligomerize on chromatin-associated RNAs^88,89^. Our data imply that physiological oligomerization of TDP-43 and FUS could limit their nuclear egress, as large oligomeric TDP-43 or FUS species are expected to poorly permeate NPCs by passive diffusion. To test this hypothesis, it will be interesting to examine whether oligomerization-deficient mutants leave the nucleus at an enhanced rate, and whether enhanced nuclear egress contributes to the reported reduction in splicing activity and enhanced cytoplasmic aggregation of oligomerization-deficient TDP-43^87^. If so, this would underscore the proposal that stabilizing physiological TDP-43 or FUS oligomers could be an attractive therapeutic strategy to counteract cytoplasmic mislocalization and aggregation of the two proteins.

For TDP-43, we furthermore found that newly synthesized RNA could retain the protein in the nucleus and limit its egress into the cytoplasm, as inhibition of transcription causes accelerated loss of TDP-43 from the nucleus. Loss of this nuclear retention mechanism could be one factor that contributes to TDP-43 mislocalization and disease progression in ALS/FTD, possibly as a consequence of the widespread RNA processing changes that occur in both diseases^3^. Interestingly, a few ALS-associated mutations in TDP-43 are located in the RRM1 domain (P112H^90^ and D169G^91^) or directly adjacent to the RRM2 domain (K263E^92^ and K267S)^93^. Moreover, stress-induced acetylation of lysine residues in RRM1 and RRM2 of TDP-43, as found in ALS spinal cord, was shown to impair RNA-binding^94^. If mutations or acetylation cause a mild RNA-binding defect (not as severe as for the F4L mutant) and thus the mutant or acetylated protein remains soluble (not oligomerized/granular as the F4L mutant), their nuclear egress might be facilitated due to reduced retention on nuclear RNA. This speculative model would be in line with a model proposed by Ling *et al*., which posits that de-regulated gene expression and disrupted protein homeostasis are intimately linked^3^. Perturbation of RNA homeostasis may cause a vicious circle that leads to further protein and RNA homeostasis defects and drives disease progression. Loss of nuclear retention of TDP-43 on newly transcribed pre-mRNAs may be part of this detrimental cycle that eventually disturbs proper TDP-43 localization and function.

## Material and Methods

### Antibodies

#### Secondary antibodies

For immunocytochemistry the following antibodies were used: Alexa Fluor® 488 Donkey anti-Goat, Alexa Fluor® 488 Donkey anti-Mouse, Alexa Fluor® 488 Donkey anti-Rabbit, Alexa Fluor® 488 Donkey anti-Rat, Alexa Fluor® 555 Donkey anti-Mouse, Alexa Fluor® 555 Donkey anti-Rabbit, Alexa Fluor® 555 Donkey anti-Rat, Alexa Fluor® 647 Donkey anti-Mouse, Alexa Fluor® 647 Donkey anti-Rabbit (Invitrogen).

For immunoblotting, the following antibodies were used: IRDye® 680RD Donkey anti-Mouse IgG, IRDye® 680RD Goat anti-Rat IgG, IRDye® 800CW Donkey anti-Rabbit IgG (LI-COR). For visualization of the the Dig(T)40 probe, FITC-labeled anti-Digoxigenin-AP, Fab fragments from sheep (Roche) was used.

#### Cloning and cDNA constructs

TDP-43 and FUS carrying mutations in putative nuclear export signals (NES) (mNES/double-mNES) were generated by QuikChange mutagenesis (Stratagene) using pcDNA6-TDP-43-V5^95^ and pcDNA3.1-hygro(−)-HA-FUS^21^ as templates. TDP-43 mNES-222 and FUS mNES-289 were used as templates to generate double-mNES mutants. TDP-43 and FUS constructs carrying mutations in the NLS (mNLS, for TDP-43: amino acids 83–85 exchanged for alanine; for FUS: P525L mutation) as well as in the putative NESs (mNLS/double-mNES) were generated by QuikChange mutagenesis (Stratagene). Constructs encoding EGFP fused to putative NES sequences were cloned by oligonucleotide annealing and HindIII/BamHI restriction digest into the pEGFP-C1 (Clontech) mammalian expression vector. To generate GCR_2_-EGFP_2_-TDP-43 and GCR_2_-EGFP_2_-FUS constructs, TDP-43 and FUS cDNAs were cloned by enzyme restriction digest into the modified pEGFP-C1 vector containing a GCR_2_-EGFP_2_ cassette^96^. FUS mRRM/mZnF (F305L/K312A/K315A/K316A/F341L/F359L/F368L/D425A/N435A/F438A/W440A/R441A/N445A) and mRGG cDNAs were commercially synthesized (Genscript) and cloned by enzyme restriction digest into the pEGFP-C2 (Clontech) mammalian expression vector. Constructs encoding flag-TDP-43 and RNA-binding-deficient mutants thereof were kindly provided by Emanuele Buratti and Francisco Baralle^51,69^. EGFP-TDP-43 (F4L) construct was generated by subcloning TDP-43-4FL into the pEGFP-C3 (Clontech) mammalian expression vector. Lentiviral constructs used for generation of stable HeLa cell lines were in pCDH-Ef1-MCS-IRES-Puro (System Biosciences). Construct encoding EGFP-histone H3.3 was kindly provided by Sandra Hake^97^. The integrity of all constructs was verified by sequencing. Oligonucleotide sequences are available upon request.

#### Stable HeLa cell lines

HeLa cells stably expressing HA-FUS were described in^98^. HeLa cells stably expressing TDP-43-V5, mCherry-TDP-43-dNLS and mCherry-FUS-P525L were generated by lentiviral transduction as described in^99^, followed by selection with puromycin (0.5 µg/ml, Sigma). Single cell clones of mCherry-TDP-43-dNLS and mCherry-FUS-P525L-expressing lines were obtained by fluorescence activated cell sorting (FACS) and expansion of mCherry + single cell clones.

#### Cell culture, transfection and drug/stress treatments

HeLa cells were cultured in Dulbecco’s modified Eagle’s medium (DMEM) with Glutamax (Life Technologies) supplemented with 10% (vol/vol) fetal calf serum (FCS, Life Technologies) and Gentamycin (10 µg/mL, Invitrogen). For the hormone-induced nuclear export assay, HeLa cells were cultured in DMEM supplemented with 10% (vol/vol) dialyzed fetal bovine serum (FBS, ThermoFisher). Transfections were carried out with Lipofectamine 2000 (Invitrogen) according to the manufacturer’s instructions. Were indicated, leptomycin B (20 nM, Santa Cruz Biotechnology) was added to the culture medium for the indicated time. Actinomycin D (5 µg/mL, Sigma) treatment was carried out for 3 hours and sodium arsenite (0.5 mM, Sigma) treatment for 30 min. Heat shock was performed by incubating cells for 1 hour in a tissue culture incubator heated to 44 °C. To induce nuclear import of GCR_2_-GFP_2_-fusion proteins, cells were incubated for 20 min with dexamethasone (5 µM, Sigma).

#### Interspecies heterokaryon assay

HeLa cells were seeded onto coverslips in a 12-well-plate and transfected with the indicated constructs. After 5 h, medium was removed and mouse embryonic fibroblasts (MEFs) were added. 24 h post-transfection, HeLa cells were fused with MEFs by a 2 min incubation with polyethylene glycol (PEG 1500, Roche) at room temperature. PEG was removed by washing 3 times in PBS supplemented with Glucose (0.1%, Merck). Post-fusion, cells were incubated in antibiotic-free DMEM in the presence of cycloheximide (75 µg/mL, ROTH) to inhibit protein synthesis and further processed by immunocytochemistry.

#### siRNA-mediated knockdown

Knockdown of Aly/REF, CRM1 and XPO5 was achieved using Dharmacon siGENOME SMARTpools composed of 4 different siRNAs. siRNA transfections were carried out with Lipofectamine 2000 at a final concentration of 25 nM siRNA. Culture medium was exchanged 5 h post-transfection and the knockdown was analyzed 72 h post-transfection by immunoblotting and immunocytochemistry.

#### NEX‐TRAP assay

The NEX-TRAP assay was performed as described^65^. Briefly, HeLa cells were co-transfected for 20 hours with the plasmid pCR3-N-HA-UL10/gM-FKBP_3_ and either pEYFP-NLS-FRB-TDP-43 or pEYFP-NLS-FRB-FUS. As control, a plasmid encoding EYFP-NLS-FRB-pUL4 was used^65^. Following 10 min anisomycin (50 µM) treatment, cells were incubated for 2 h with rapamycin (150 ng/ml, + RAP) or without rapamycin (−RAP) in the presence of anisomycin. Subsequently, gM was visualized by immunocytochemistry, EYFP-tagged proteins were visualized directly and nuclei were visualized by DAPI staining.

#### Poly(A)+RNA *in situ* hybridization

All reagents were treated with 0.1% diethyl pyrocarbonate (DEPC, Sigma). Cells grown on 12 mm coverslips were fixed for 15 min at room temperature in 4% formaldehyde in PBS, permeabilized on ice for 5 min in 0.5% Triton X-100 in PBS and equilibrated on ice for 5 min with 2× SSC (Ambion) and 25% formamide in PBS. Cells were hybridized for 2 h at 37 °C with 1 ng/ml digoxygenin labeled oligo(dT) (40mer, Eurofins) in 2× SSC, 1 mg/ml tRNA, 0.02% bovine serum albumin (BSA), 2 mM ribonucleoside vanadyl complex (RVC, Sigma), 25% formamide, 5% dextrane sulfate. Afterwards, cells were washed 2× with 2× SSC/25% formamide in PBS, followed by a washing step with 0.5× SSC in PBS and once with 0.2% Triton X-100 in PBS. Subsequently, immunocytochemistry was carried out using a FITC-coupled anti digoxygenin Fab fragment (1:300, Roche) for detection of the labeled oligonucleotide and specific antibodies for the indicated proteins, respectively.

#### Immunocytochemistry

All steps were carried out at room temperature. Cells were fixed for 10 min in 4% formaldehyde in PBS, permeabilized for 5 min in 0.2% Triton X-100 supplemented with 50 mM NH_4_Cl and blocked for 30 min with 0.1% saponine in PBS supplemented with 5% goat or donkey serum. Primary antibodies (see Table 1) and secondary antibodies were diluted in 0.1% saponine in PBS and were applied for 45 min each and washed with 0.1% saponine in PBS. Coverslips were mounted onto glass slides using ProLong Gold Antifade Reagent with DAPI (Invitrogen) and dried at room temperature overnight.

**Table 1:** List of primary antibodies used.

Antigen	Species, antibody name	Source
Aly/REF	Mouse (monoclonal, 11G5)	Abcam
Coilin	Mouse (5P10)	Kind gift of A. Lamond (Dundee, UK)
β-Actin	Mouse (monoclonal, AC-74)	SIGMA-Aldrich
CRM1/Exportin-1	Goat	refs^94,102^
flag	Rabbit (F7425)	SIGMA-Aldrich
FUS	Rabbit (A300-302A)	Bethyl
FUS	Mouse (4H11)	Santa Cruz
GAPDH	Rat (10F4)	Kind gift of Helmholtz Center Munich Antibody Core Facility
G3BP1	Rabbit (13057-2-AP)	Proteintech
EGFP	Rabbit (A1122)	Thermo Scientific
gM	Rabbit	Gift of Thomas Mettenleiter, refs^102,103^
HA	Rat (monoclonal, 3F10)	Roche
Histone H3	Rabbit	Abcam
human-hnRNP-C1/C2	Mouse (monoclonal, 4F4)	Abcam
mCherry	Rabbit (ab167453)	Abcam
p62/SQSTM1	Rabbit (polyclonal, PM045)	MBL International Corporation
TDP-43 (405–414)	Rabbit (TIP-TD-P09)	Cosmo
TDP-43 (404–413)	Rat (6D6)	Kind gift of Helmholtz Center Munich Antibody Core Facility
V5	Rabbit (polyclonal, AB3792)	Merck
XPO5	Mouse (monoclonal, ab57491)	Abcam

#### Image acquisition and quantification

Images were acquired with an inverted Zeiss Axio Observer.Z1 wide-field fluorescence microscope with a 63/1.4NA oil immersion lens and an AxioCam506 and analyzed with Zen software (Zeiss). If necessary for better print quality, images were processed by linear enhancement of brightness and contrast. Images presenting the NEX-TRAP results were taken using a confocal laser scanning microscope (LSM710, Zeiss) and processed using Adobe Photoshop CS3 by linear enhancement of brightness and contrast.

For quantification of mean fluorescence intensity (MFI) in the nucleus and cytoplasm, images were imported in the public-domain software Fiji^100^. In ImageJ, ROIs corresponding to nuclei were identified by DAPI staining using the wand tool and MFI in the EGFP channel in nuclear ROI was determined. A band around the nucleus (band size = 2 µm) was set as ROI corresponding to the cytoplasm and MFI of the EGFP signal in the cytoplasmic ROI was measured. For each construct, 40 cells from one representative experiment were analyzed.

#### Preparation of RIPA lysates

HeLa cells were harvested in trypsin/EDTA (Sigma) and washed twice with ice-cold PBS. Cells were lysed in ice-cold RIPA buffer (50 mM Tris-HCl pH 8.0, 150 mM NaCl, 1% NP-40, 0.5% sodium deoxycholate, 0.1% sodium dodecyl sulfate) supplemented with complete protease inhibitor cocktail (Roche). Lysates were sonicated and and protein concentrations were determined by BCA protein assay (Pierce).

#### Nuclear/cytoplasmic fractionation and nuclear extraction

HeLa cells were harvested in trypsin/EDTA (Sigma) and washed twice with ice-cold PBS. Cells were incubated in mild cell lysis buffer (20 mM Tris, pH 7.4, 10 mM KCl, 3 mM MgCl_2_, 0.1% NP-40, 10% Glycerol and complete protease inhibitor cocktail (Roche)) for 10 min on ice and centrifuged (2000 g) for 10 min at 4 °C to pellet nuclei. Nuclear proteins that are insoluble in mild cell lysis buffer remain in the pellet fraction (nuclear fraction, N), whereas soluble nuclear proteins are extracted into the supernatant (cytoplasmic fraction, C). 4× SDS-PAGE buffer was added to the cytoplasmic fraction, the nuclear fraction was resuspended in the same volume of mild cell lysis buffer and 4× SDS-PAGE buffer was added. Equal volumes of cytoplasmic and nuclear fractions were analyzed by SDS-PAGE and immunoblotting with the indicated antibodies.

#### SDS-PAGE and immunoblotting

4× SDS-PAGE buffer was added to samples and samples were boiled for 5 min at 95 °C. Proteins were separated by SDS-PAGE gel and transferred to a nitrocellulose membrane (Amersham™ Protran™ 0.2 µm NC, GE Healthcare Life Sciences). The membrane was blocked in blocking buffer (Tris-buffered saline supplemented with 0.1% Tween-20 (TBS-T) and 5% milk powder) and incubated with the indicated primary antibodies (see Table 1) and secondary antibodies diluted in blocking buffer, followed by 3 washes in TBS-T. Bound antibodies were visualized using the LI-COR fluorescent immunoblotting system (Odyssey CLx Imaging system).

#### Western blot quantification and stastitical analysis

Immunoblots from three independent cell fractionation experiments were analyzed. Mean grey values (MGV) in regions-of-interest (ROI) bordering the protein bands were measured by Fiji/ImageJ software. MGVs were calculated and converted into cytoplasmic to nuclear ratios (c/n). Means of the three independent experiments were calculated and standard error of the mean indicated by error bars. The one-way ANOVA with Dunnett’s multiple comparison test (Fig. 6B) as well as the t-test for paired samples (Fig. S5C) were used for statistical analysis.

#### Fluorescent Loss In Photobleaching (FLIP) analysis

HeLa cells were cultured in µ-Dish 35 mm, high Glass Bottom (ibidi) and transiently transfected with EGFP-TDP-43or EGFP-histone H3.3 as a control. 24 h post-transfection cells were either left untreated or were treated with actinomycin D (5 µg/ml) for 3 hours. Images were acquired with an inverted Zeiss Axio Observer.Z1 microscope with a 63/1.4NA oil immersion lens equipped with a confocal spinning disc (CSU-X1, Japan) and a Rapp OptoElectronic laser scanning device (UGA-42, Germany). Before bleaching, 5 images were taken in streaming mode with a 488 nm 50 mW SD laser; the same setting were used to acquire an image after bleaching. For bleaching, 3 circular ROIs of 6 µm each were repeatedly photobleached in the cytoplasm using a 473 nm diode laser (DL-473/75, Rapp OptoElectronic) with full laser power with an iteration of 100 and duration of 200 ms per bleach event. An image was acquired before and after each bleach event with 15 s intervals between each bleach event.

The fluorescence loss of a defined area in the nucleus of the bleached cell was measured over time, and corrected for bleaching by acquisition and background noise as follows using the Fiji/ImageJ macro “TimeSeries Analyzer”:

$${\rm{I}}({\rm{t}})=[{\rm{ROI}}1({\rm{t}})-{\rm{ROI}}3({\rm{t}})]/[{\rm{ROI}}2({\rm{t}})-{\rm{ROI}}3({\rm{t}})].$$

ROI1 is defined as the average grey value of an area in the nucleus of the cells which cytoplasm was repeatedly photobleached. A corresponding area of a non-photobleached cell in the same field of view served as control for bleaching due to image acquisition and is represented by the average grey value of ROI2. ROI3 is the defined average grey value of the background. Furthermore, average grey values were normalized to the mean grey value of the 5 pre-bleach images (set to 1).

## Electronic supplementary material

Supplementary Information
