# Supplementary material for: Nuclear egress of TDP-43 and FUS occurs independently of Exportin-1/CRM1
Source: Sci Rep. 2018 May 4;8:7084. doi: 10.1038/s41598-018-25007-5 (PMC5935713; doi:10.1038/s41598-018-25007-5)

Supplementary information

Nuclear egress of TDP-43 and FUS occurs independently of Exportin-1/CRM1

Helena Ederle, Christina Funk, Claudia Abou-Ajram, Saskia Hutten, Eva B.E. Funk, Ralph H. Kehlenbach, Susanne M. Bailer, Dorothee Dormann

A

TDP-43	WT sequence	Mutated sequence
mNES-222	IPKPFRAFAF	IPKPARAAAF
mNES-239	IAQSLCGEDLII	IAQSACGEDAII
Double-mNES	IPKPFRAFAF<br>IAQSLCGEDLII	IPKPARAAAF<br>IAQSACGEDAII

FUS	WT sequence	Mutated sequence
mNES-289	VQGLGENVTI	VQGAGENATI
mNES-301	VADYFKQIGI	VADYAKQAGI
Double-mNES	VQGLGENVTI<br>VADYFKQIGI	VQGAGENATI<br>VADYAKQAGI

B

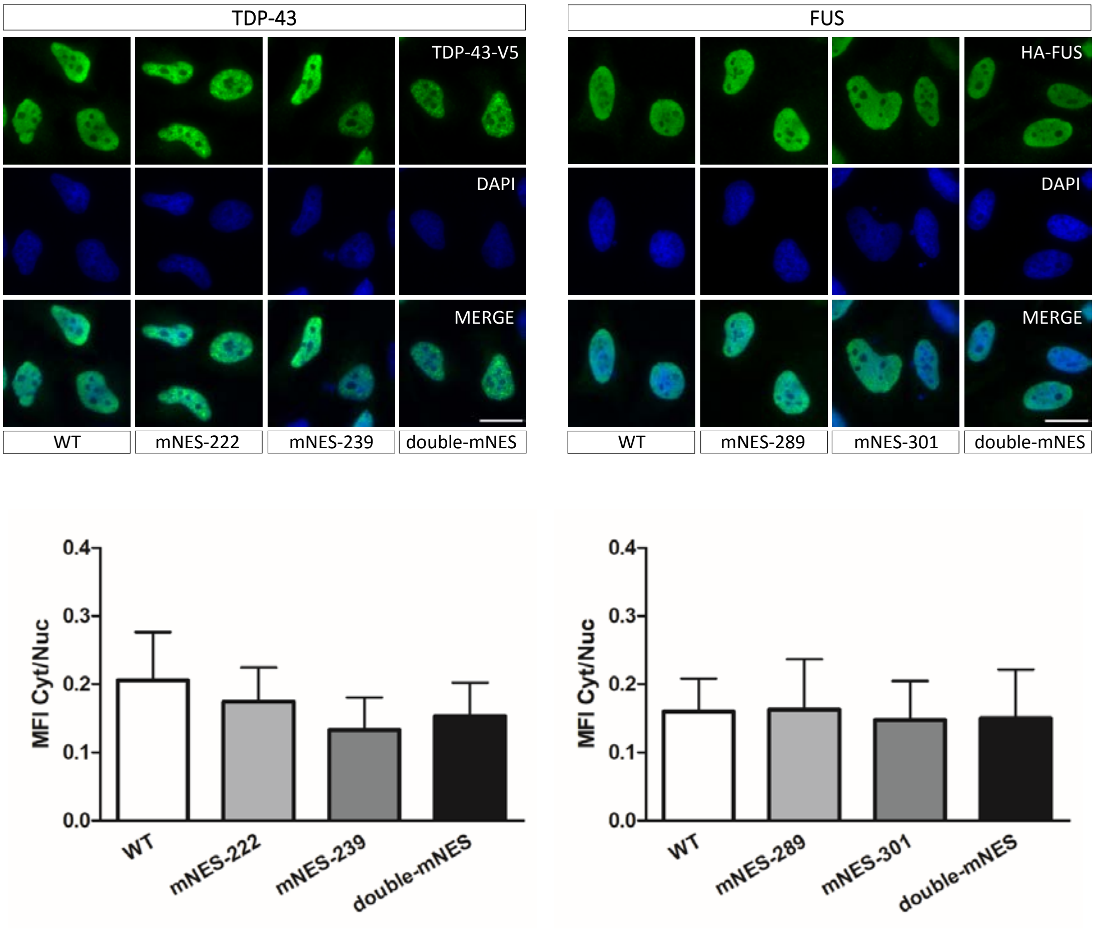

Supplementary Figure S1.

A. Table shows amino acid sequences of putative CRM1-dependent NESs predicted to be present in TDP-43 and FUS, hydrophobic amino acids are underlined. In a functional CRM1-dependent NES, exchange of hydrophobic amino acids for alanine (A) should abrogate CRM1-binding and nuclear export activity. We therefore exchanged two hydrophobic residues in each predicted NES to alanine (A). Mutated residues in mNES constructs are underlined and highlighted in red.

B. V5-tagged TDP-43 or HA-tagged FUS wild-type (WT) or with mutations in one or both predicted NESs (mNES or double mNES) were expressed in HeLa cells and their localization was examined after V5- or HA-immunostaining (green) and DAPI staining (blue) by fluorescence microscopy. Scale bars: 20 μm. Bar graphs below images show quantification of the mean fluorescence intensity (MFI) in the cytoplasm (Cyt) / nucleus (Nuc) in 40 cells from one representative experiment out of three, error bars indicate SD.

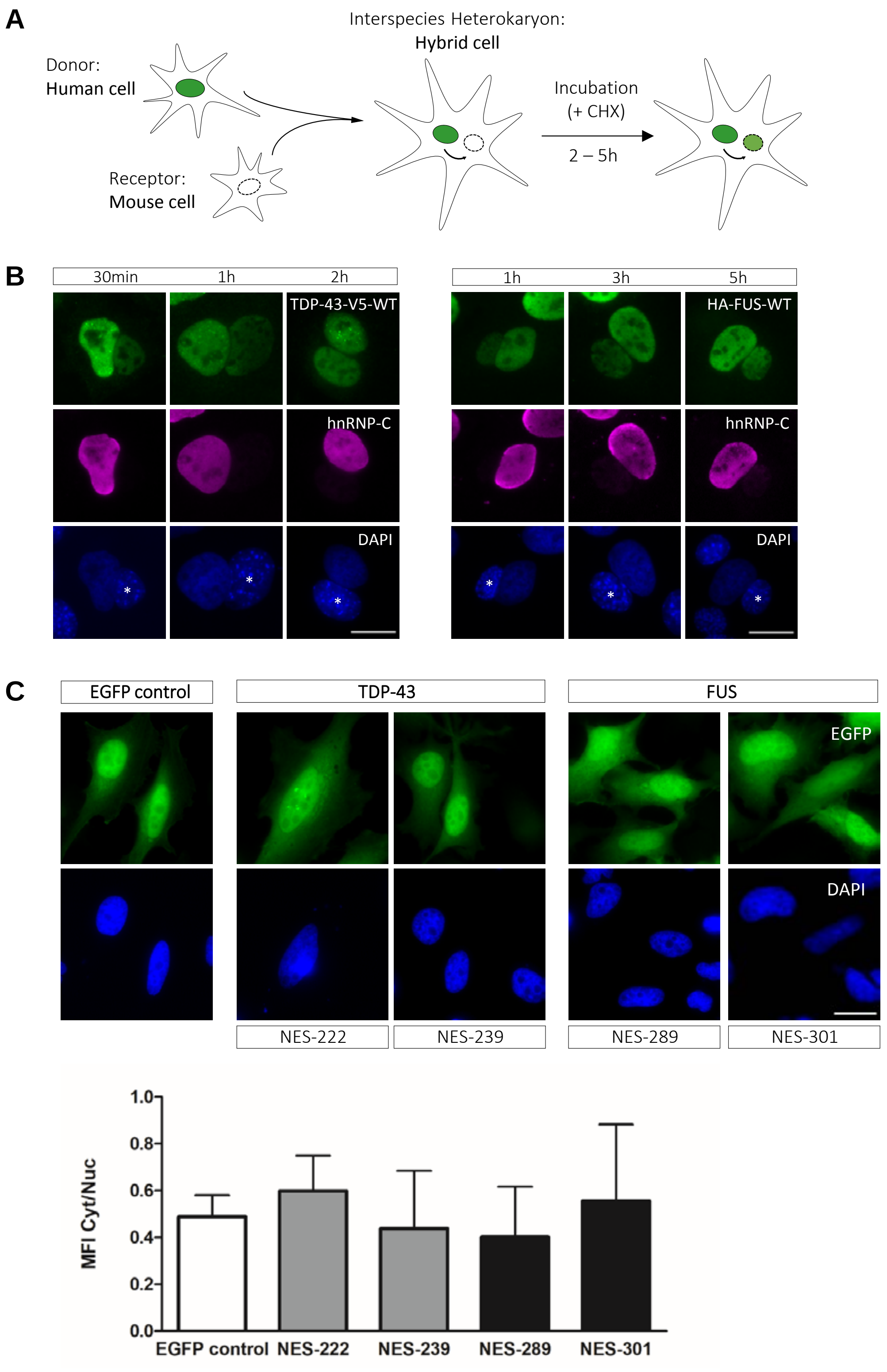

Supplementary Figure S2.

A. Schematic diagram of the interspecies heterokaryon assay. Cells of two different species (e.g. human and mouse) are fused to generate hybrid cells with two nuclei from different species ("interspecies heterokaryons"). Heterokaryons are incubated for 2-5 hours in the

presence of the protein synthesis inhibitor cycloheximide (+CHX) to suppress *de novo* protein synthesis in the cytoplasm. Shuttling of a protein from one nucleus to the other (here: human to mouse) indicates nuclear export of the protein.

B. HeLa cells stably expressing V5-tagged TDP-43 or HA-tagged FUS were fused to MEFs and heterokaryons were analyzed by immunocytochemistry at various time points after fusion (30 min, 1h, 2h for TDP-43; 1h, 3h, 5h for FUS). The TDP-43 signal in mouse nuclei (marked with an asterisk in the DAPI channel) reaches a maximum 2h after fusion. FUS accumulates in mouse nuclei more slowly and reaches a maximal signal intensity 5h post-fusion. This indicates that TDP-43 and FUS shuttle between the nucleus and cytoplasm and that TDP-43 shuttles more rapidly than FUS. Scale bars: 20 μm.

C. EGFP or EGFP fused to the indicated predicted NESs of TDP-43 or FUS were expressed in HeLa cells and their localization was examined by fluorescence microscopy by imaging EGFP fluorescence (green) and DAPI staining (blue). Scale bar: 20 μm. Bar graph show a quantification of the mean fluorescence intensity (MFI) in the cytoplasm (Cyt) / nucleus (Nuc) in 40 cells from one representative experiment out of three, error bars indicate SD.

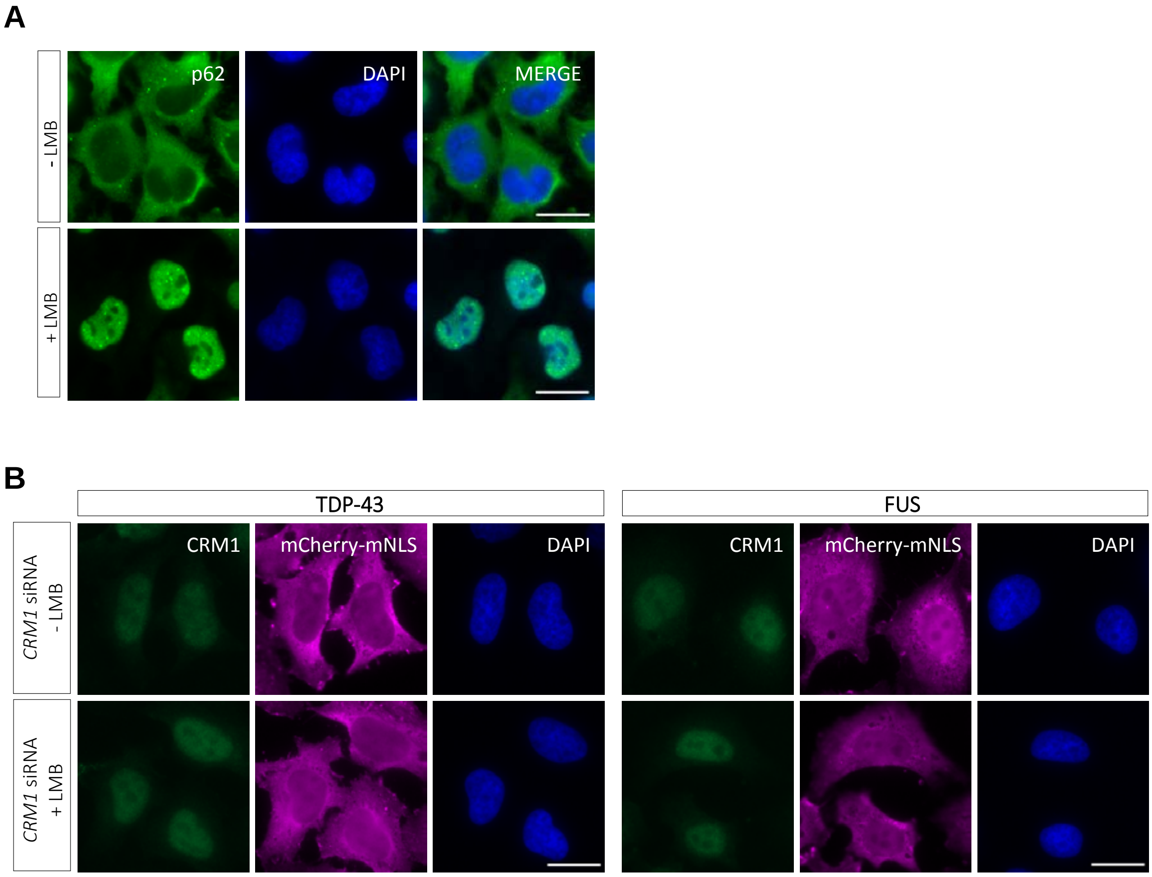

Supplementary Figure S3.

A. To demonstrate activity of the CRM1-specific inhibitor leptomycin B (LMB), HeLa cells were treated for 2.5 h with LMB (20 nM) and localization of a known CRM1 cargo, p62/SQSTM1, was analyzed by p62 immunostaining (green), DAPI staining (blue) and fluorescence microscopy. LMB treatment causes an accumulation of p62/SQSTM1 in the nucleus, demonstrating efficient inhibition of p62/SQSTM1 nuclear export. Scale bars: 20 µm.

B. Combined inhibition of CRM1 activity using CRM1-specific siRNA and LMB treatment. HeLa cells stably expressing NLS mutant mCherry-tagged TDP-43 or FUS were transfected with CRM1-specific siRNA and 3 days post-transfection, cells were treated for 2.5 hours with LMB (20 nM). Cells were stained with an CRM1-specific antibody (green), mCherry-specific antibody (magenta) and DAPI (blue) and were imaged by fluorescence microscopy. Even the combined treatment does not affect localization of NLS mutant mCherry-TDP-43 or FUS. Scale bars: 20 µm.

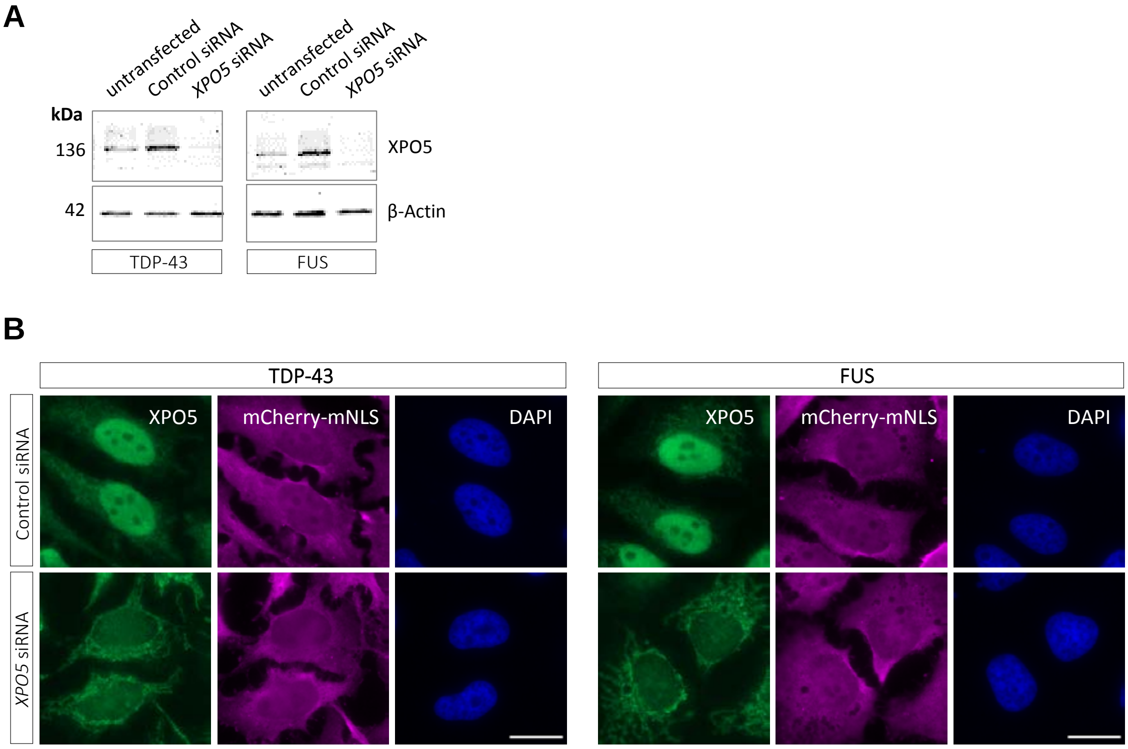

Supplementary Figure S4

HeLa cells stably expressing mCherry-TDP-43-mNLS or mCherry-FUS-mNLS were transfected with control or Exportin-5 (*XPO5*)-specific siRNA. 3 days post-transfection, XPO5 levels in total cell lysates were analyzed by Western blotting, β-actin served as a loading control (**A**), demonstrating efficient reduction of XPO5 protein levels. In parallel, cells were stained with an XPO5-specific antibody (green), mCherry-specific antibody (magenta) and DAPI (blue) and were imaged by fluorescence microscopy (**B**). Silencing of Exportin-5 does not affect localization of NLS mutant reporter proteins, indicating that nuclear export of TDP-43 and FUS is independent of Exportin-5.

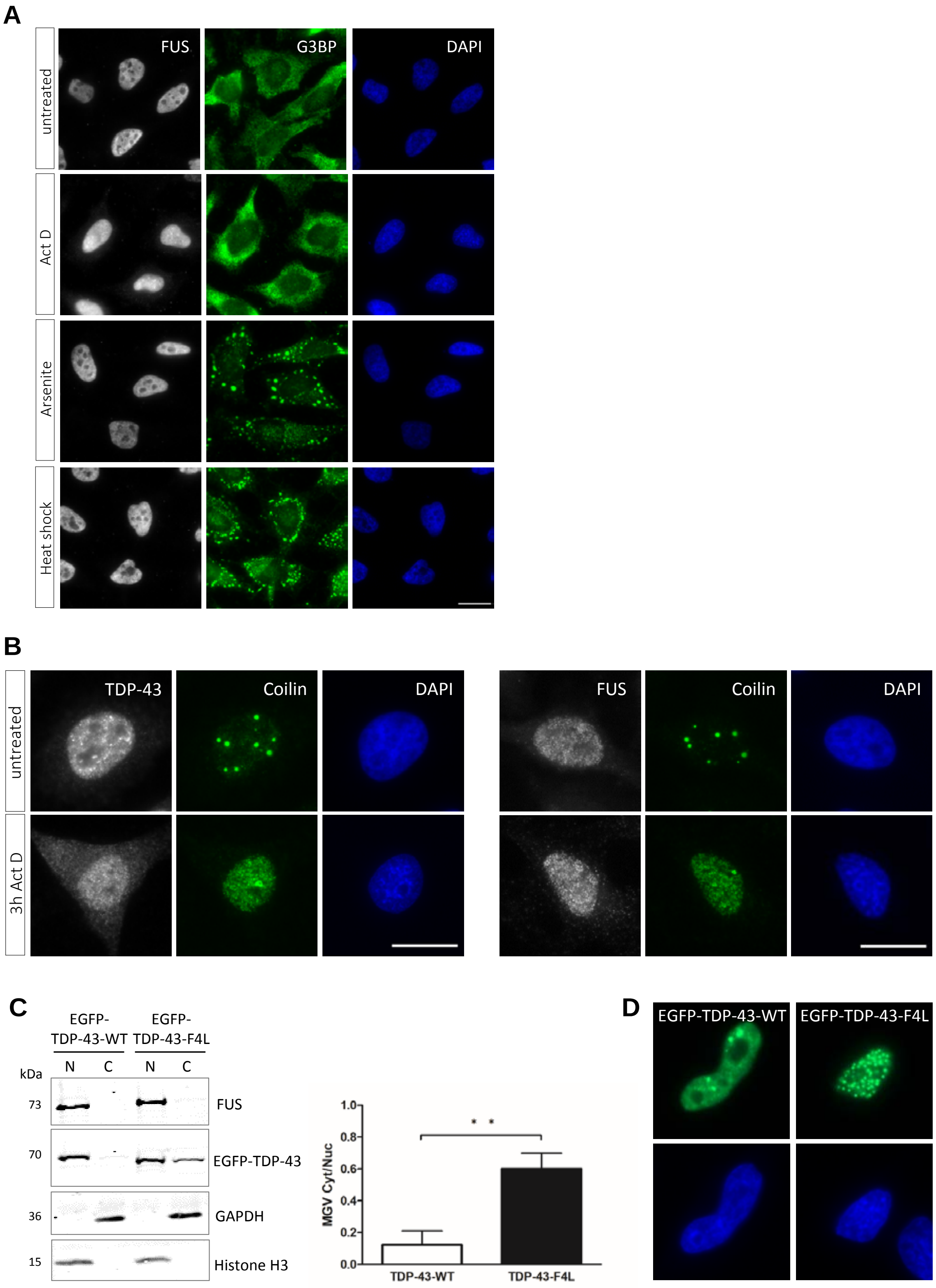

Supplementary Figure S5

A. HeLa cells were either left untreated or treated with Actinomycin D (Act D, 5 µg/ml for 3h), arsenite (0.5 mM for 30 min) or heat shock (44°C for 1h) and localization of TDP-43 and stress granule formation were examined after immunostaining with antibodies specific for

FUS (white), the stress granule marker G3BP1 (green) and DAPI staining (blue). FUS remains predominantly nuclear under all stress treatments. Scale bar: 20 µm.

B. HeLa cells were incubated with Act (5µg/mL) for 3 h, stained for TDP-43 or FUS (white), Coilin (as a positive control for transcriptional inhibition, green) and DNA (DAPI, blue) and imaged by fluorescence microscopy. Act D treatment causes a partial cytosolic relocalization of TDP-43, while FUS remains predominantly nuclear. Coilin staining indicates transcriptional inhibition, as it is known to redistribute into nucleolar caps upon transcriptional inhibition[97]. Scale bars: 20 µm.

C. HeLa cells expressing EGFP-tagged TDP-43-WT or the RNA-binding deficient F4L mutant were separated into nuclear (N) and cytoplasmic (C) fractions, analyzed by SDS-PAGE and immunoblotting with the indicated antibodies. RNA-binding deficient EGFP-TDP-43 is partially extracted from nuclei and thus appears in the cytoplasmic fraction, whereas EGFP-TDP-43-WT is not extracted and is predominantly found in the nuclear fraction. Bar graph (right) shows a quantification of TDP-43 immunoblot signals. Mean grey value (MGV) of bands in cytoplasmic/nuclear fraction of three independent experiments are shown, error bars represent SEM. **p-value ≤ 0.01 calculated with t-test for paired samples.

D. HeLa cells were transiently transfected with EGFP-TDP-43-WT or the RNA-binding deficient 4FL mutant and localization was analyzed by fluorescence microscopy. Both proteins are nuclear, the 4FL mutant displays nuclear foci/granules, as previously reported[67].

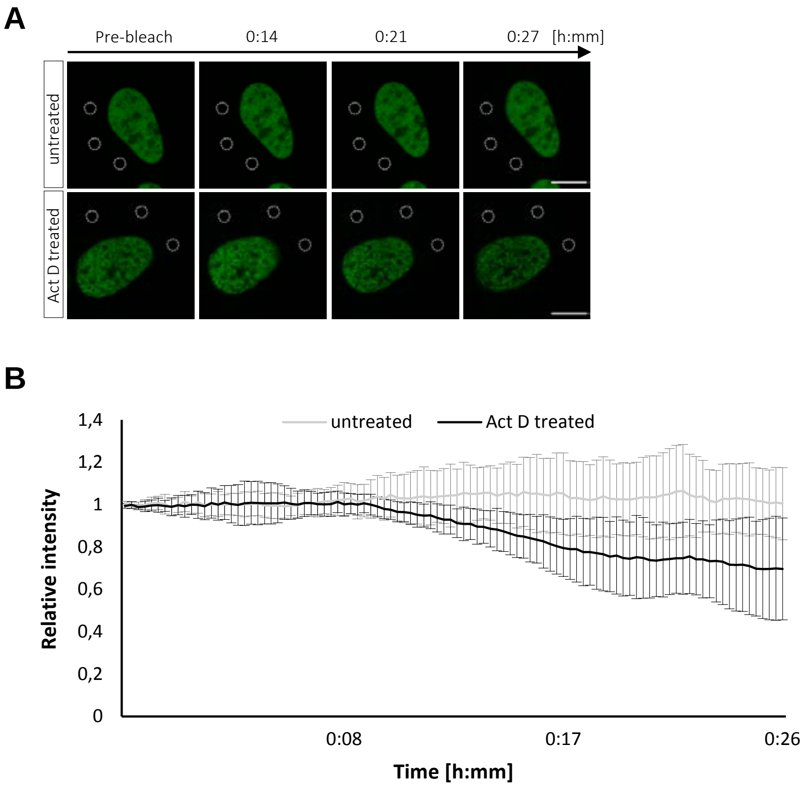

Supplementary Figure S6

A. Representative images of untreated and 3h Act D-treated EGFP-Histone H3.3-expressing cells recorded during FLIP analysis. At later time points, Act D-treatment causes a slight loss of the nuclear EGFP-signal compared untreated cells. Scale bars: 10 µm.

E. Relative fluorescence intensity of the nuclear EGFP-Histone H3.3 signal measured during FLIP analysis in untreated and 3h Act D-treated HeLa cells. Compared to EGFP-TDP-43, EGFP-histone H3.3 shows only a slightly decreased relative fluorescence intensity of the nuclear EGFP-signal in Act D-treated cells.

Full-length blots (for assembled/cropped blots shown in Fig. 2, 3 and 6):

Figure 2D Raw Data:

U = untransfected, C = siRNA control, KD (knockdown) = CRM1 siRNA

anti-CRM1 Western Blot:

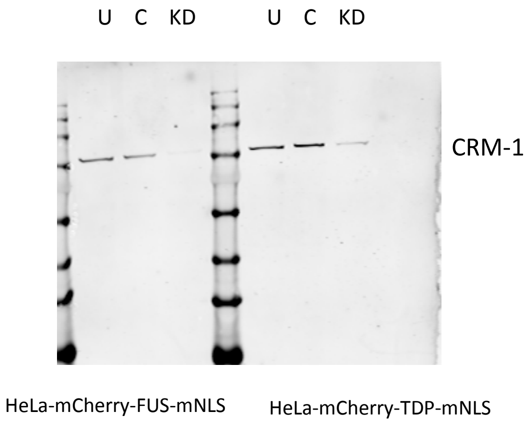

anti-beta-Actin Western Blot:

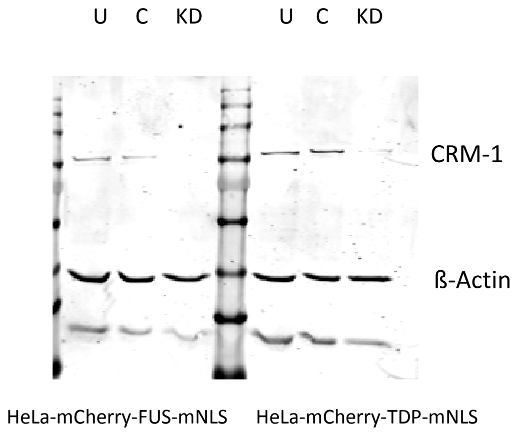

<u>Figure 3C Raw Data</u>

U = untransfected, C = siRNA control, Aly = Aly siRNA, NXF1 = NXF1 siRNA

HeLa-mCherry-TDP-mNLS cells:

One blot developed with anti-Aly/REF & anti-beta-Actin antibodies, right lane (NXF1-knockdown) cropped off:

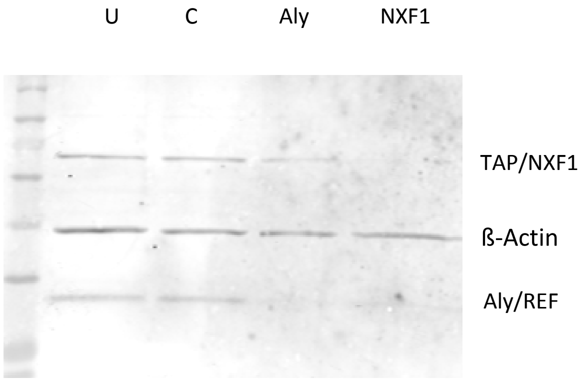

HeLa-mCherry-TDP-mNLS

HeLa-mCherry-FUS-mNLS cells:

One blot developed with anti-Aly/REF & anti-beta-Actin antibodies, right lane (NXF1-knockdown) cropped off:

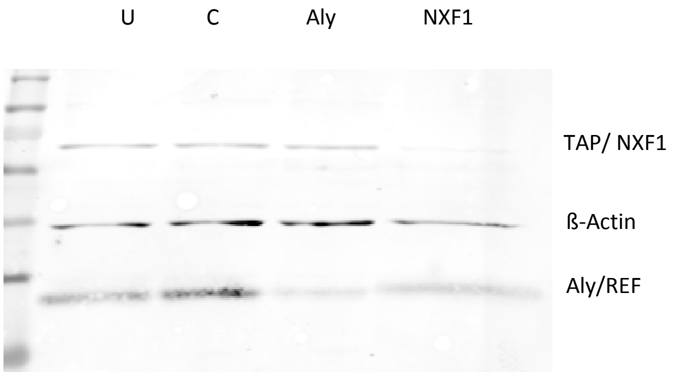

HeLa-mCherry-FUS-mNLS

Figure 6B Raw Data

Assembled from four blots:

First blot developed with anti-TDP-43 & anti-GAPDH antibodies, second blot developed with anti-GAPDH antibody, third blot developed with anti-FUS and fourth blot developed with anti-Histone H3

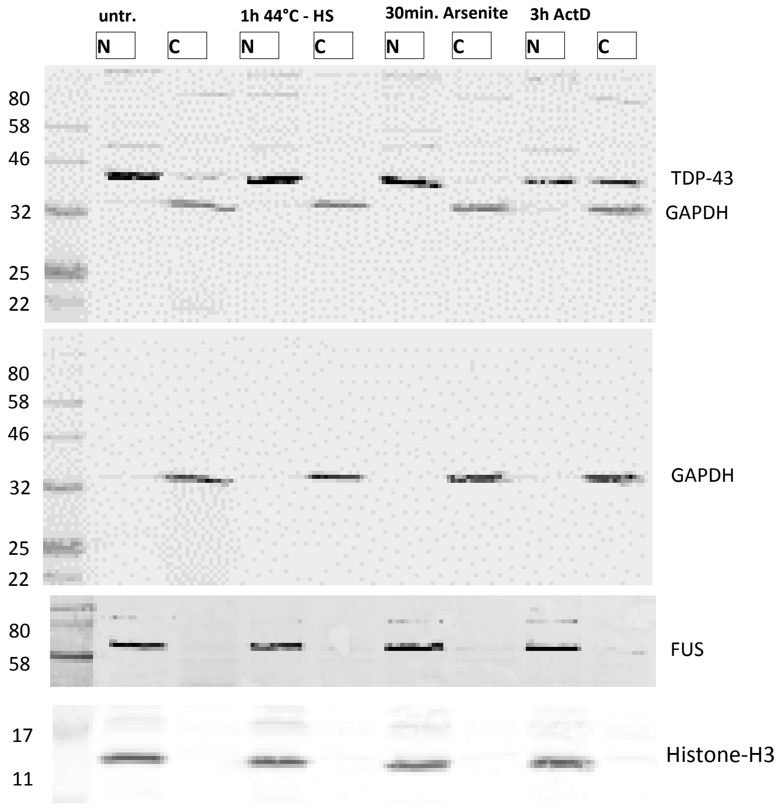

Supplementary Figure S4A Raw Data

U = untransfected, C = siRNA control, XPO5 = XPO5 siRNA

Assembled from 2 blots detecting indicated proteins

anti-XPO5 Western Blot

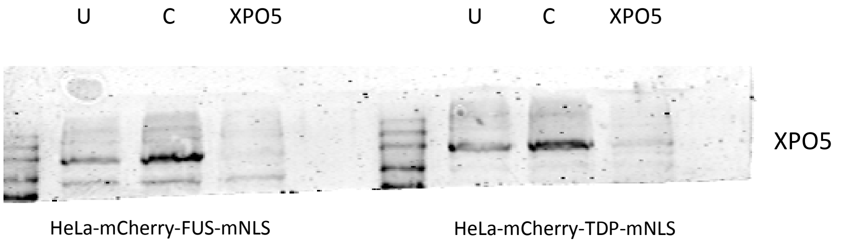

anti-beta-Actin Western Blot for

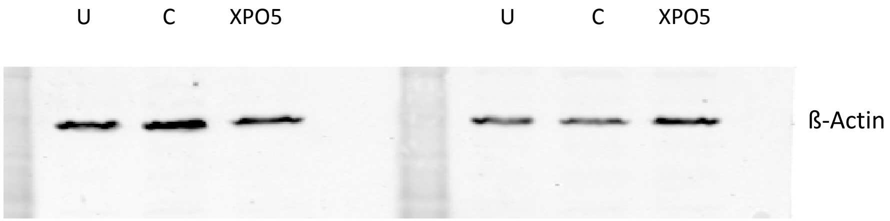

Supplementary Figure S5C Raw Data

Assembled from 4 blots detecting indicated proteins

anti-FUS Western Blot:

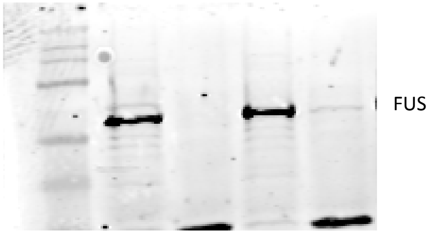

anti-EGFP Western Blot: (detecting EGFP-TDP-43-WT and -4FL):

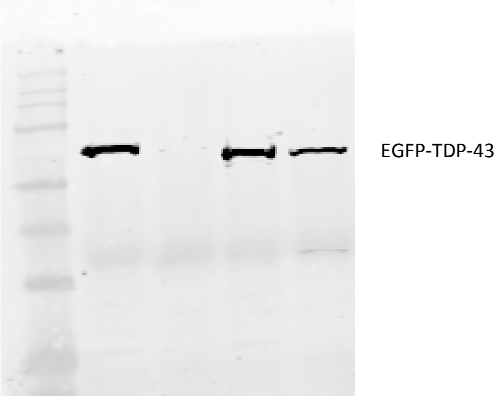

anti-GAPDH Western Blot:

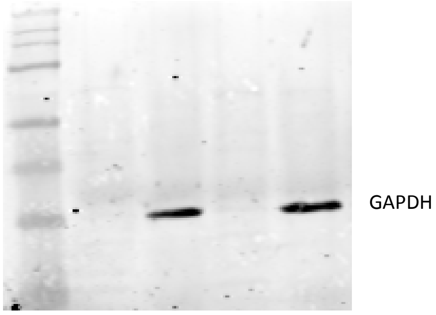

anti-Histone H3 Western Blot:

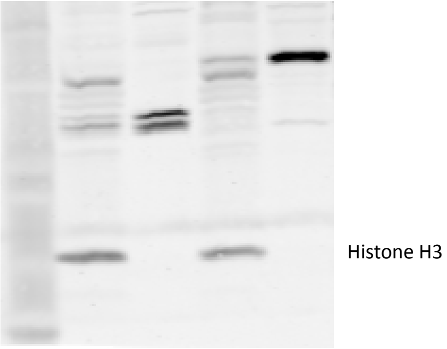

Supplement: Supplementary file 1 — Supplementary Information [file 41598_2018_25007_MOESM1_ESM.pdf]
